# Lignins as Promising Renewable Biopolymers and Bioactive Compounds for High-Performance Materials

**DOI:** 10.3390/polym15153177

**Published:** 2023-07-26

**Authors:** Cornelia Vasile, Mihaela Baican

**Affiliations:** 1 Romanian Academy, “P. Poni” Institute of Macromolecular Chemistry, Physical Chemistry of Polymers Department 41A Grigore Ghica Voda Alley, RO700487 Iaşi, Romania; 2 “Grigore T. Popa” Medicine and Pharmacy University, Faculty of Pharmacy, Pharmaceutical Sciences I Department, Laboratory of Pharmaceutical Physics, 16 University Street, RO700115 Iaşi, Romania; m_pascu2000@yahoo.com

**Keywords:** lignin, raw material, bioactive compound, nanoparticles, nanofibers, hydrogels, green composites, applications

## Abstract

The recycling of biomass into high-value-added materials requires important developments in research and technology to create a sustainable circular economy. Lignin, as a component of biomass, is a multipurpose aromatic polymer with a significant potential to be used as a renewable bioresource in many fields in which it acts both as promising biopolymer and bioactive compound. This comprehensive review gives brief insights into the recent research and technological trends on the potential of lignin development and utilization. It is divided into ten main sections, starting with an outlook on its diversity; main properties and possibilities to be used as a raw material for fuels, aromatic chemicals, plastics, or thermoset substitutes; and new developments in the use of lignin as a bioactive compound and in nanoparticles, hydrogels, 3D-printing-based lignin biomaterials, new sustainable biomaterials, and energy production and storage. In each section are presented recent developments in the preparation of lignin-based biomaterials, especially the green approaches to obtaining nanoparticles, hydrogels, and multifunctional materials as blends and bio(nano)composites; most suitable lignin type for each category of the envisaged products; main properties of the obtained lignin-based materials, etc. Different application categories of lignin within various sectors, which could provide completely sustainable energy conversion, such as in agriculture and environment protection, food packaging, biomedicine, and cosmetics, are also described. The medical and therapeutic potential of lignin-derived materials is evidenced in applications such as antimicrobial, antiviral, and antitumor agents; carriers for drug delivery systems with controlled/targeting drug release; tissue engineering and wound healing; and coatings, natural sunscreen, and surfactants. Lignin is mainly used for fuel, and, recently, studies highlighted more sustainable bioenergy production technologies, such as the supercapacitor electrode, photocatalysts, and photovoltaics.

## 1. Introduction

At present, in all countries, priority is given to the good quality of the environment and its preservation. Because of the exhaustion of fossil product resources, both research and industry units search for environmentally friendly materials manufactured independently on petroleum products, which means, in other words, the development of new biomaterials for the possible replacement of existing products, like conventional plastics. New materials will be developed by combined green chemistry and engineering technology [1]. Nowadays, bioplastics represent annually only around one percent of the total plastic produced, but it is expected that the demand for bioplastics will increase [2,3,4]. As a consequence, numerous studies have been conducted to develop natural alternatives, starting from biodegradable, renewable resources, which are safer and cleaner for the environment, as precursor materials instead of petroleum and also using innovative technological approaches developed to control pollution [5].

The biomass of living organisms in the global biosphere is expressed in gigatons of carbon, with 1 Gt C = 10^15^ g of carbon [6]. Plant biomass as a renewable resource constitutes ~80% of the 550 GtC of total biomass, as an abundant available resource in the biosphere [6]. The biomass distribution on Earth indicates that the dominant kingdom primarily comprises terrestrial plants with ≈450 Gt C; animals with 2 Gt C are mainly from the seas and oceans, while bacteria (≈70 Gt C) and archaea, as microorganisms that define the limits of life on Earth, (≈7 Gt C), are predominantly located in deep subsurface environments. The terrestrial biomass is about two orders of magnitude higher than the aquatic biomass and it contains more consumers than producers [7,8]. The enormous potential of biomass as a petroleum alternative is exemplified by its availability as in the United States alone; it accounts for ~1 billion tons/year of dry biomass [9]. Thus, the demand for development of environmentally friendly and sustainable production/consumption/waste management systems for both plastics and biodegradable materials has changed in recent years because of modifications in consumer habits; preferences for single-use products, fresh food, and ready-to-eat packed food; awareness of the need for a healthy life and environment; and the growing need for fabricating packaging, medical devices, healthcare products, and others in a more environmentally friendly way. Biomass-based materials are extensively studied, with recent research being focused on them because they present biodegradability, compostability, low carbon footprint, and recyclability and appropriate physical, mechanical, and barrier properties [10].

Plant lignocellulosic biomass is one of the most abundant raw materials and sustainable resources in the world, with a production rate of 2 × 10^11^ tons/year. Lignocellulosic biomass (LCB), a complex heterogeneous mixture comprising carbohydrate polymers, namely cellulose (40–45% *w*/*w*) and hemicellulose (25–35% *w*/*w*) and lignin (15–30 wt%), includes agricultural residues, energy crops (temperate grasses), and wood residues. Its abundance in nature could potentially solve the problem of the rapidly depleting resources if it is used as renewable resource or valorized to higher value materials. LCB is by far the largest proportion of the natural material available on terrestrial earth for sustainable production of energy and chemicals. Global demand for biofuels is set to grow by 41 billion liters, or 28%, over 2021–2026 [11,12,13]. Conventionally, they are used in second-generation biofuel plants, for production of ethanol, diesel, methane, etc. The Energy Security and Independence Act of 2007 called for the annual production of nearly 80 billion L of second-generation biofuels by 2022, which implies roughly 62 million tons of lignin byproduct.

Lignin, an organic polymer is potentially an import market product as a raw material for chemicals and life science industries. Lignin, the second-most abundant renewable biopolymer on Earth, is one of the largest natural renewable sources of aromatic structures and the second-largest renewable source of carbon, with a heating value similar to carbon. Its aromatic compounds have multiple special chemical properties and show important bioactive effects. Some studies on the energy need in bioethanol plants show that the energy content of lignin is higher than the need for ethanol production; this will always lead to an energy excess which can be used for other purposes like external uses or as a chemical raw material. A computer simulation has been developed to evaluate the efficiency of lignin separation from lignocellulosic raw materials, like straw and spruce, for bioethanol production and purposes other than energy [14].

Lignin exhibits impressive properties, including good mechanical and physicochemical properties, low weight, antioxidant and antimicrobial properties, and excellent thermal stability, and it can undergo a range of modifications to tailor or impart special characteristics, such as improved compatibility and processability, that explain the interest in extending its applicability in various domains. Since lignin has intra- and intermolecular hydrogen linkages, it exhibits thermoplastic properties at low temperatures, being useful for lignin-derived biobased polymers and film materials, and can also serve as a thermoset at high temperatures (T > 200 °C) because of its substantial crosslinking. At elevated temperatures, significant degradation could arise [15]. Therefore, lignin possesses huge potential for the production of a variety of materials because of its high carbon content, low cost, and bio-renewability [16]. It can be considered an excellent alternative for developing novel green functional and sustainable materials as plastic substitutes, considering its availability, excellent environmental friendliness, ecological adaptability, degradability, reinforcing ability, etc. The interest in lignin from the scientific and industrial communities increased over the last decade because of the growing concern over climate change and the need to decrease industrial pollution [17]. Therefore, lignin is a viable alternative to fossil nonrenewable resources for energy, chemicals, polymers, and various materials [18,19,20].

The main technical lignin resources are pulp and paper mills and emerging cellulosic biorefineries, as well as agricultural and forestry residues. The annual production of industrial lignin is approximately 1.8 × 10^9^–2 × 10^10^ tons, which is mainly obtained as a byproduct from the pulp or paper industry and emerging cellulosic biorefineries, while a cellulosic ethanol plant can generate 100,000–200,000 tons lignin/year [21]. Presently, only ~2% of lignin as a byproduct is recovered, while 98% is burned for energy or dumped in landfills, this alternative creating a major disposal issue [22]. Lignin as a byproduct of pulp and paper production processes is available commercially. The major fraction of lignin is used in boilers to generate steam and satisfy the heating requirements of the biomass processing plants (thermal recycling) and recover inorganics. The small fraction of lignin (~2%) is used for manufacturing of vanillin, dispersants, animal feed, cement fillers, carbon fibers, agglomerates, adhesives, renewable materials, etc. Therefore, it is still underutilized. Biorefineries generate green energy through well-established processes such as fermentation of corn, sugar cane, and wheat to obtain bioethanol, and also biodiesel can be produced by the transesterification of rapeseed and soybean oil. The sustainability of these processes is debatable because the utilization of the edible crops competes with food production. To avoid a potential increase in food price and deforestation, a new generation of biorefineries is being developed by utilizing nonedible lignocellulosic biomass. There are several types of technical lignins, such as kraft lignin, lignosulfonate, alkali lignin, acid hydrolysis, steam explosion lignin, and organosolv lignin (see below). Recently, new treatments have been applied, such as steam-assisted or solvent-assisted biomass fractionation, which produce lignins for targeted applications. Annually, lignin production with the kraft process, with approximately 130–170 kton, produces the largest amount of lignin compared to other production processes.

Lignin valorization involves both the use and application of the whole polymer and the exploration of low-molecular-weight oligomers [23,24,25,26,27,28].

Lignin applications in the field of polymer materials started in 2010 and include the use of lignins as dispersants, phenolic resins, components of thermoplastics, polyurethane (PUR) foams, epoxy resins, and asphalt or concrete modifiers [29], where lignin could act as a superplasticizer. Lignin as a dispersant improves viscosity and fluidity of bioasphalt, concrete, drilling mud for oil recovery, and dyes for textile and agricultural chemicals [30]. Modified lignins are used as concrete modifiers as lignin-derived plasticizers [31].

Lignins provide a physical-strength-forming water-conducting vascular network and protect plants against attacking organisms and insects. Lignin also acts as a natural additive/bioactive compound. The role of natural additives and other components with a concentration smaller than 15% raises interest because they assure quality and functionality of many products. Their role in various materials as natural additives, including lignin and lignans, is to improve physicochemical properties: mechanical, barrier, sealing, optical, biodegradability, hydrophobicity, sensory, etc. Bioactive compounds (BCs) or “active ingredients” used in many advanced materials for food packaging, medical devices, cosmetics, etc., induce desired functionalities such as antimicrobial, antifungal, and antioxidant activities. Bioactive compounds can be used as antimicrobial agents to inhibit the harmful activity of bacteria, fungi, and viruses, being very interesting not only in terms of their biological activities but also for their biocompatibility, renewability, and biodegradability, etc. Active compounds are very important in tailoring the advanced polymeric material properties, assuring their applications in many fields especially if they are used with a corresponding concentration and they show a modulated controlled release. They can be developed and implemented in industries as adhesives, biosurfactants, antimicrobial agents in packaging and textiles, antioxidants, adhesives, anticorrosion agents, carbon fiber or carbon black, cosmetics, reinforcing agents, hydrogels, phenolic resin, flame retardants, polyurethane, foam composites, compatibilizers, and new biomedical materials. Functionalities of lignin and its derivatives include binding, dispersing, crystal growth modification, emulsion stabilization, antioxidant and antimicrobial activities, UV-absorbing capabilities, biocompatibility, and low cytotoxicity, confirming its role as a bioactive compound. Soda lignin plays a significant role in antifungal and antibacterial activity since it contains various functional groups, i.e., carboxylic acid and hydroxyl groups [32]. In new bioasphalts, a part of bitumen is replaced by renewable resources as demonstrated in a study in the Netherlands, where 50% bitumen was replaced by three lignins, namely soda, kraft and hydrolysis, with the technology being in TRL5-7 stage [33]. Many applications are in the development stage; therefore, the studies are still in the patent stage.

Applications have been found for nanostructures based on natural biopolymers including lignin in multiple fields, including bioactive/smart packaging, cosmetics, medical fields, pharmaceutics, drug delivery systems, textiles, food, adhesives, plastics, paper, and the environment [34].

Lignin-based biomaterial delivery systems, consisting of microparticles, nanoparticles, hydrogel, bio(nano)composites, and blends, offer the possibility to improve the efficiency of drug delivery, as they protect drugs from degradation, increase their absorption and transport, and, in the case of tailor-designed nanoparticles, favor targeting [35,36,37,38]. Therefore, lignin is a multipurpose/multifunctional raw material with important roles/applications in the various fields.

The review papers published in this field are related to such applications as production of chemicals and fuels [7,9]; lignocellulosic bioethanol plants [14]; lignin biopolymers [18,19,20,23]; agricultural applications of lignin [25]; bio-oil, bio-binder, and bio-asphalt materials [30]; valorization in medicine, cosmetics, and environmental remediation [35]; food packaging [36]; and r additives [39]. This review highlights the recent developments in lignin use, both as a raw material and bioactive compound in new types of high-performance materials, emphasizing on the multiple applications of lignins and their role in sustainable development to evidence their role as promising renewable biopolymer sources and bioactive compounds. It is divided into ten main sections, starting with an outlook on lignin diversity; main properties and possibilities to be used as a raw material for fuels, aromatic chemicals, plastics, or thermoset substitutes; and new developments in the use of lignin as a bioactive compound and in nanoparticles, hydrogels, 3D-printing-based lignin biomaterials, new sustainable biomaterials, and energy production and storage. In each section are presented recent developments in the preparation of lignin-based materials, especially the green approaches to obtaining nanoparticles, hydrogels, and multifunctional materials as blends and bio(nano)composites, by selecting the most suitable lignin type for each category of the envisaged products/applications and the main properties of the obtained newly developed lignin-based materials, etc. Different application categories of lignin within various sectors, which could provide completely sustainable energy conversion, such as in agriculture and environment protection, food packaging, biomedicine, and cosmetics, are described. The medical and therapeutic potential of lignin-derived materials is evidenced in applications such as antimicrobial, antiviral, and antitumor agents; carriers for drug delivery systems with controlled/targeting drug release; tissue engineering and wound healing; and coatings, natural sunscreen, and surfactants. Lignin is mainly used for fuel, and, recently, studies highlighted more sustainable bioenergy production technologies, such as the supercapacitor electrode, photocatalysts, and photovoltaics.

## 2. Lignin Structure and Properties

**Figure 1:**
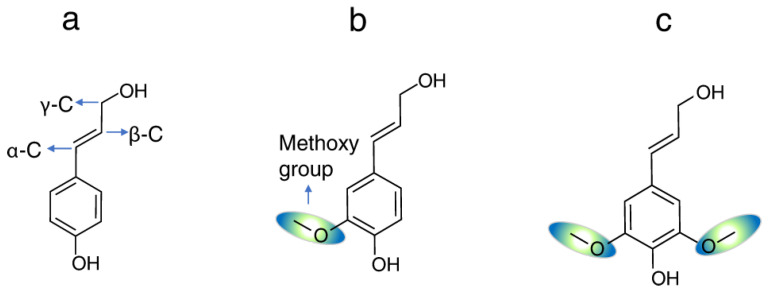
Primary monomer units used for lignin sequence generation: (**a**) p-coumaryl alcohol or H monomer, (**b**) coniferyl alcohol or G monomer and (**c**) syringyl alcohol or S monomer.

Lignin is a three-dimensional, amorphous, randomly crosslinked network consisting of both aliphatic and aromatic components of methoxylated and hydroxylated phenylpropanoid units, the main ones being p-hydroxyphenyl (H), guaiacyl (G), and syringyl (S) units, derived from p-coumaryl, coniferyl, and sinapyl alcoholic precursors, respectively (Figure 1). Lignin deconstruction results in these three major aromatic motifs, as fundamental monolignols of lignin. Their composition and properties depend on different extraction processes as well as the deconstruction method and source [39]. These three units are repeatedly linked by various chemical bonds, such as β-O-4, 4-O-5, β-5, β-1, β-β, 5-5, spirodienone, and dibenzodioxocin [40].

Based on the different number of the main lignin units, wood can be classified into three groups, namely softwood, with a high preponderance of guaiacyl lignin (G); hardwood, with a certain composition of G and syringyl units (S) and a very small proportion of p-coumaryl alcohol or p-hydroxyphenyl units (H); and herbaceous, containing mainly G units and a smaller proportion of H units [41].

### 2.1. Analytics and Structure

Lignin must be analyzed to identify its composition and molecular constitution, to determine the proportions of its main building blocks (guaiacyl, syringyl, and p-hydroxyphenyl), and, based on these possible reaction strategies, to utilize some chemical/biological pretreatments and to achieve a final conversion to useful desired compounds [42]. All protocols for lignin characterization have been summarized by the International Lignin Institute (ILI) and have been reviewed during July 2009 and updated in 2023 [43]. Each type of lignin should be characterized from structural and morphological perspectives and with respect to various properties to evidence its particularities required to define the application domain [44,45,46,47,48]. Pyrolysis–gas chromatography/mass spectroscopy (Py-GC/MS) provides a “structural fingerprint” of H-, G-, and S-substituted phenols as constituents of lignin. Lignin with a large number of G units has a more condensed structure because the aromatic C5 position is available for coupling, resulting in strong C-C bonds, while S-units are mainly linked by more labile ether bonds at C4 positions of aromatic rings. The average molecular weight of lignin and its distribution affects biomass recalcitrance, lignin valorization and mechanical properties, and melt viscosity, etc. It is determined by gel-permeation chromatography (GPC) or size-exclusion chromatography. Spectroscopic methods are used to assess the detailed structure of polymers [46]. Thermal behavior of lignin as a special biopolymer indicates that Tg of lignin varies with water content and varies between 95 and 162 °C depending on lignin type and applied pretreatment [49,50]. Thermal decomposition temperature depends on the heating rate and atmosphere. Lignin decomposition begins at about 280 °C with a maximum rate occurring between 350 and 450 °C and the completion of the reaction at 450 °C and 500 °C [51].

Natural polyphenols, such as lignins, lignans, and tannins, represent important structural materials in the support tissues of plants and valuable products of secondary plant metabolism that have a fundamental role in different stages of plant life.

Lignans are compounds with a 2,3-dibenzyl-butane structure. Lignans possess a steroid-like chemical structure, being defined as phytoestrogens. They are minor constituents of many plants, including higher plants (gymnosperms and angiosperms), such as whole grains, legumes, vegetables, and seeds, with the highest concentrations of lignans found in flax seed [52,53], where they form the building blocks for the formation of lignin (as distinguished from lignans) found in the plant cell wall. The bioactive properties of lignans as human health-promoting molecules include a lowered risk of heart disease, menopausal symptoms, osteoporosis, and breast cancer.

### 2.2. Technical Lignins

Cellulose/lignin is obtained from wood and non-wood plants as lignocellulosic biomasses, lignin being the second-most abundant biopolymer of terrestrial biomass. Lignin is an important source for biobased products; its valorization can also contribute to reducing greenhouse gas (GHG) emissions. As already mentioned, pulp and paper mills and second-generation biorefineries produce large quantities of low-value technical lignin as a byproduct.

Several physical, chemical, and physicochemical fractionation methods of lignins from LCB have been developed. The commonly used lignin extraction processes are the kraft process (kraft lignin or alkali lignin), sulfite process (lignosulfonates), and organosolv process (organosolv lignin), while for medical uses the enzymatic hydrolysis process (hydrolytic lignin) is preferred. Each method alters the chemical structure and molar mass of native lignin in a different way, thus conferring different characteristics that result in various levels of performance as a substrate for chemical/biochemical transformations. Technical lignins have particular chemical structures with respect to their functional groups and properties, and therefore they differ by structure, composition, molecular weight, water solubility, and purity, with covalently bonded sugars residues or sulfur, degree of condensation, and content of functional groups, such as –OH aliphatic or aromatic, methoxyl, and carboxyl groups (Table 1).

**Figure 2:**
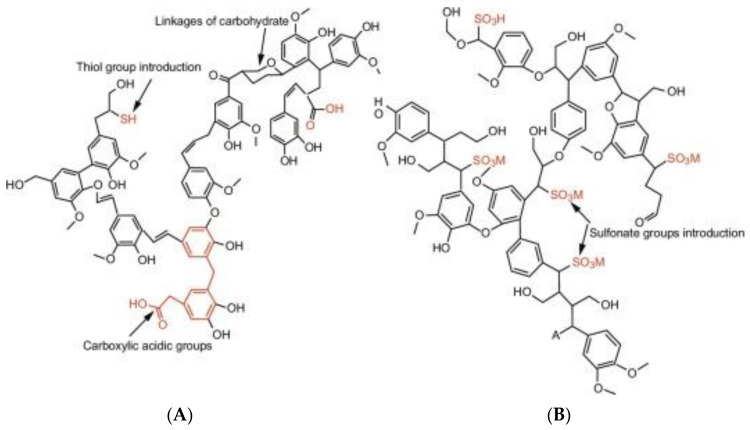
Structures of kraft pine lignin (**A**) and lignosulfonate lignin (**B**) [56].

The difference between technical lignins is the uniqueness of the functional groups present in each of them [57]. Differences in molecular structure are also found in lignins resulting/extracted from different sources (plant family and species, their culture conditions, part of plant, age, climate, etc.) and extraction procedures [58,59], as evidenced for kraft lignin and lignosulfonates in Figure 2. They can contain different amounts of carboxyl, carbonyl, phenolic, and aliphatic hydroxyl groups. The properties and main applications vary with lignin type; soda lignin possesses superior mechanical properties, being suitable for the production of phenolics, animal nutrition, dispersants, and polymer synthesis, while organosolv lignin exhibits improved water retention ability and is sulfur-free, also being of high purity, quality, and chemical reactivity, which makes it ideal for direct use or upgradation into high-value chemicals. Lignosulfonates are randomly branched polyaromatic polyelectrolytes that are water-soluble and exhibit surfactant-like behavior. They are mostly used as emulsifiers, antistatic agents, dispersants, and in the electronics sector.

Following the sulfite pulping process, where sulfite base can differ (e.g., Na, Ca, NH_4_^+^, Mg) and aqueous SO_2_ (pH 1–2) is used for delignification of the lignocellulosic biomass, the differences include changes in molecular weight, solubility, variations in aromatic content, and the presence of impurities, ash, and sulfur. Lignosulfonate has mostly been used in manufacturing wearable electronics mainly due to high methoxy and carbonyl groups on the lignin backbone. Lignosulfonate has been produced at a rate of 1 million tons/year.

In soda and alkali lignin, sulfur is absent, but the content of methoxy groups is very high at 10–19%, enabling their use in the synthesis of phenolic resins and making it possible to obtain bioplastics and biocomposites. Compared with kraft and lignosulfonate, organosolv lignin is more hydrophobic and has a low glass transition, being considered a high-value biochemical for obtaining elastomers, polyesters, polyurethanes, carbon fibers, binders, and coating resins. The kraft method is recognized as the primary source of commercial-grade lignin [57].

Recently developed processes to separate kraft lignin are LignoBoost (1997), Domtar (2003), and LignoForce (2008). Soda and alkali lignins were obtained by GreenValue SA, India, in 2003 and by North Lignin Chemical in 2010. In 2019, in Limeira, São Paulo, Suzano began a pioneering initiative in lignin and its derivatives—ECOLIG0 as the first plant for lignin derived from 100% certified Eucalyptus planted and grown from selected clones and harvested in Brazil with a production capacity of 20,000 tons/year. The LignoBoost plant for lignin separation is now operational in Stora Enso’s Sunila mill, Finland, separating lignin from black liquor by treating the black liquor with carbon dioxide and a strong acid. This lignin extraction process replaces a large amount of natural gas with dried lignin, so the carbon dioxide emissions are reduced. The LignoBoost plant is the second commercial-scale manufacturing center in the world. Lignin powder can be used as a raw material, or it can be converted into other materials and products.

Acid-hydrolyzed lignin has a wide molecular weight and high methoxy group content, being suitable for synthesis of various types of polymers. Avantium (NL, 2000) and ST1 (FI, 1995) produced such lignin. New producers of organosolv lignins are Biomass Industrial Company CIMV, France (2006); the Fibria/Lignol Pilot plant 2010; Fortum/Chempolis (FI) 2009; and Frauhofer LEUNA Park (DE). Steam explosion lignin resembles native lignin due to mild extraction conditions being preferred in the phenolic resin synthesis [56].

Networking groups of scientists and experts devoted to lignin valorization for large/industrial application of materials containing lignin, production of chemicals, etc. have been assembled, such as the International Lignin Institute (1991, Lausanne, Switzerland), Lignin Club Ecosystem, LignoCOST, LIGNOVAL, and Wageningen UR Lignin Platform.

### 2.3. Other Sources of Lignin

Agricultural waste contains approximately 80 wt% of lignocellulosic materials. It includes straw, husk, stalks, stover, and cobs as the largest known sources of wasted lignin. As recent preoccupations have shifted toward greener solutions, agricultural crop residues have been considered highly valuable as bioenergy and biorefinery materials [60,61]. The extraction of lignin is one of the major barriers in the reclamation and reuse of lignin from waste. The following principles have been considered: (1) make full use of the lignin from the waste source, (2) effectively avoid the loss of carbon, and (3) decrease the impact on the environment caused during combustion. Natural resources, such as plants, animals, and the aquatic medium, are envisaged, but if huge quantities are used, competition with the food industry will result. To avoid this competition and to direct main activities toward sustainability and environment protection, the valorization of byproducts and biowastes is exploited. Valorization of the residual products or byproducts and biowaste as sources of biopolymers and BC is a feasible strategy for ecofriendly, biobased, good-performance materials and could be an important solution enabling a transition from a polluting, fuel-based to a circular, biobased economy. Food-grade and suitable materials acting as matrices in various materials and ingredients are abundant in biowastes and byproducts. Better-controlled composition and properties of the obtained products is necessary.

## 3. Biobased Products from Lignin

Lignin can be a matrix and/or additive in various fields. Common uses of lignin-based compounds are as flame retardants, wood adhesives, flocculants, lubricants, dispersant media, prebiotics, animal feed supplements, food additives, natural antioxidants and antimicrobial agents, reinforcement for polymers, components in biocomposite/nanocomposites materials, adsorbents for heavy metals and toxic organic compounds from the environment, biobased thermoplastics, platform chemicals, BTX, hydrocarbons, carbon fibers, aromatic monomers, renewable chemicals, smart materials, biofuels, and battery electrode fuels, including production of carbon materials and energy storage materials. The vast recent research developments in the application of lignin consist of creating lignin nanoparticles, hydrogels, and lignin for 3D printing, biocomposites, surfactants, electrodes for energy storage devices, and chemical deconstruction of lignin for the production of platform chemicals. As final polymeric compounds, phenolic resins and polyurethanes have already been mentioned. For specific uses, certain qualities/characteristics of lignin are required. A low content of inorganic and hemicellulose moieties is preferable for lignin-based fuels. High S lignin content is desirable for lignin as a food additive and in food applications due to higher hydroxyl substitution in the lignin units. Low molecular weight and high presence of carboxyl (C=O) and alkyl hydroxyl (OH–) groups are suitable properties for lubricants in polyethyleneglycol. For the production of aromatic compounds, a low degree of condensation (or a high degree of hydration) is necessary [62]. The utilization of low-quality/high-quantity lignin (e.g., kraft lignin) to produce high-added value compounds is achieved via two methods: by deconstruction (sometimes named depolymerization) into smaller fragments and specific chemical modification/functionalization. Valorization of lignin as a raw material to obtain oils or fine chemicals by deconstruction involves several routes of reactions/techniques, such as [63] thermochemical/pyrolytic methods; direct liquefaction at elevated temperatures [64] producing complex product mixtures; base/acid-catalyzed techniques such as reductive, oxidative, and solvolytic chemical modifications occurring in organic solvents in the presence of acids, bases, metals, or ionic liquids [65]; and biological or enzymatic degradation [66]. All of these methods should be followed by upgrading [67].

However, the valorization of lignin to high-performance and cost-competitive materials remains a challenge. It has been demonstrated that the biopolymers better adhere to ”green design” principles in comparison to petroleum counterparts, but their properties rank below those of several common petrochemical plastics (e.g., polyolefins) with respect to environmental impact considering a full life-cycle analysis [68]. Moreover, lignin valorization has encountered a series of constraints related to heterogeneous polymeric nature/composition, intrinsic recalcitrance, strong smell, dark color, some problems encountered in lignocellulose fractionation, recalcitrance to depolymerization/deconstruction, and a complex mixture of aromatic compounds resulting during degradation, etc. However, lignin-based compounds are excellent candidates for greenhouse gas diminution in comparison to many petroleum-based chemicals [69,70]. Selected criteria to assess the sustainability of lignin valorization by applying such techniques include waste generation, atom and energy efficiency, usage of safer solvents, biocatalytic methodology, environmental factor (E factor), and atom economy. The greenness evaluation provides a quantitative base for strategic decisions. It was concluded that future collaboration between industry, academia, and policy regulators is needed to assess optimal processing and manufacturing routes of lignin-based polymers and to realize the potential of lignin in this regard. A suitable valorization of lignin waste streams from the pulp and paper industry and biorefinery processes could be a crucial step for the development of a circular sustainable economy.

### 3.1. Aromatic Chemicals

There are many opportunities for producing fine chemicals and pharmaceuticals from biomass. Due to the high content of aromatics, lignin is a prospective natural source to produce a range of aromatic chemicals, such as phenol, guaiacol (1-hydroxy-2-methoxybenzene), and vanillin (4-hydroxy-3-methoxybenzaldehyde). The difficulties in lignin use to obtain aromatic chemicals or building blocks chemicals mainly derive from its complex variability in chemical composition, molecular weight, and impurities, depending both on natural source and methods used in biorefineries/separation. Lignin as a feedstock for producing fuels, chemicals, and other materials is already an advanced-stage process. Technical lignins result in large quantities as low-cost industrial resources directed to obtain renewable aromatic chemicals. The conversion routes of technical lignins and other LCBs to biobased aromatics are (non-catalytic) lignin pyrolysis, direct hydrodeoxygenation, and hydrothermal upgrading. The products generated are mixed oxygenated aromatic monomers, light organics, heavy organics, and biochar. Hydrodeoxygenation was found to be the most promising [71] because of the highest return on investment. Hydrothermal liquefaction of lignin occurring in near-critical water conditions produces a bio-oil that is suitable for co-feeding into a petroleum refinery hydrotreatment unit to obtain water-soluble organics, gaseous products, and char. Lignin decomposes into phenolic compounds as the main products when the process is conducted under high-temperature and supercritical water conditions, which may be followed by liquid–liquid extraction and hydrotreatment to produce benzene, toluene, ethylbenzene, and xylenes (BTEX) compounds..Coproduction of BTEX and biofuel did not reduce the biofuel cost. Lower bio-oil oxygen content and decreased capital and operating costs are necessary to make hydrothermal liquefaction-based fuels competitive with fossil fuel-based options [72,73]. At low temperatures (<300 °C), methoxyphenols are the most commonly obtained monomers, whereas, when the reaction is developed at high temperatures (T > 300 °C), catechol and alkylcatechols are the main products [74,75].

Oxidative depolymerization using heterogeneous catalysts is a viable upgrading option to produce aromatic monomers. Base-catalyzed depolymerization/deconstruction is extensively studied because of its simplicity and efficiency. It has been established that the cleavage of ether bond of lignin leads to phenolic oil generation, but unstable reaction intermediates participate in repolymerization reactions through reformation of carbon–carbon bonds.

Production of *Benzene*, *Toluene*, *and Xylenes (BTX*) from lignin, as coproducts of biorefining, started in 1970, and an increasing interest still exists, as evidenced by the high number of publications and patents [76,77,78]. The processes applied to low-quality lignins are hydrothermal liquefaction operating in critical water conditions, fast pyrolysis (T > 600 °C), catalytic fast pyrolysis from agroindustrial biomass blended with PVC, and the use of NaZSM-5 and HZSM-5 as catalysts [79]. Reductive catalytic fractionation generates phenolic fractions, which, via deoxygenation, lead to specific chemicals such as BTX. Catalytic fast pyrolysis of biomass indicates that oxygen-containing compounds decreased, whereas aromatics increased, and, at the same time, the increase in the formation of mono-aromatics and reduction in polyaromatic hydrocarbons occurred. All mentioned technologies are in R&D stages, and many improvements are still being studied. Olive pomace and almond shell valorization through this process is inexpensive. BTX yield varies between 10 and 30%. BTX are used to obtain both polymers and other chemicals with a wide range of applications, such as healthcare and pharmaceutics, the automotive industry (parts of cars), packaging, electronics, textiles, sport, and construction. Benzene is used to obtain polystyrene (PS) and toluene for polyurethanes and as an additive in gasoline and para-xylene for production of polyethyleneterephthalate (PET) and polyamides, etc. The increasing use of biobased BTX is expected to open new market opportunities, and their quantity will increase in the next 10 years by 50%, reaching 220 million tons in 2030 (in accordance with EC directives).

### 3.2. Phenolic Compounds

Catalytic reductive deconstruction of lignin is a promising and effective method for valorization to obtain phenolic monomers [80,81]. As an example, liquefaction/fragmentation of lignin using biochar-derived, activated-carbon-supported metal catalysts as bimetallic catalyst Ni-Co/AC (5 to 20 wt %), and various solvents (water, ethanol, methanol) was tested for alkali lignin at 260 to 300 °C for a 15 min reaction time. The process, using an ethanol solvent system at 280 °C, gave the highest bio-oil yield (72.0 wt %) [82]. In the liquefaction/fragmentation of lignin using chloride (MClx) and Pd/C, biochar-derived, activated-carbon-supported catalysts produced a phenolic monomer (namely 85.6% lignin liquefaction with 35.4% phenolic monomer yield [83]) and vanillin (34.8%) [82].

Phenolic compounds are known as natural compounds with several beneficial biological effects, such as antioxidant and anti-carcinogenic activities. Their antioxidant activity is dependent on their structure, as it is affected by the reduction in or inhibition of free radicals via transfer of a hydrogen atom (hydrogen atom transfer—HAT) from their hydroxyl group or by single-electron transfer (SET). The reaction of a phenolic compound with a peroxyl radical (ROO•) involves the transfer of the hydrogen cation from the phenol to the radical, forming a transition state of a H-O bond [84]. Phenolic compounds are useful for various industrial applications, such as in foods, cosmetics, and pharmaceutics. The major drawbacks are their low bioavailability and pH, temperature, and light. Nanotechnology and lipidic encapsulation systems are alternatives to overcome these limitations, to protect molecules from external factors, and to improve their bioavailability. The loading of polyphenols into lipid nanocarriers (NCs) is an efficient way to increase their bioavailability, for reducing degradation, and protecting antioxidant activity of the polyphenols [85,86].

Because of the multiple possibilities in conduction biorefinery and lignin valorization, the simulation of biorefinery processes for the design of manufacturing processes to obtain value-added chemicals from lignocellulosic resources has been conducted, involving phenols from lignin [87], biochemicals [88], and eugenol and phenolic products [89].

Due to the complex structure of lignin, it cannot be degraded by commonly known degradation methods. Specific microorganisms, such as bacteria and certain fungi such as wood-rotting *Basidiomycetes* fungi can degrade lignin by producing enzymes both in the presence and absence of oxygen; under this effect, lignin can be broken down into simpler compounds and used as a carbon source for growth. The main microbial degraders of lignin are white-, brown-, and soft-rot fungi and soil fungi, which produce several extracellular enzymes, such as laccases, lignin peroxidases (LiP), manganese-dependent superoxide dismutase peroxidase (MnP), dye-decolorizing peroxidase (DyP) multicopper oxidase, and polyphenol oxidoreductases and versatile peroxidase, which are effective for lignin degradation via the generation of free radicals. The lignolitic enzymes from bacteria and fungi are useful in delignification of biomass and depolymerization of lignin [90]. Similarly, white-rot fungi such as *Ceriporiopsis subvermispora*, *Phlebiopsis gigantea*, and *Coriolus hirsutus* are used in the biological pulping process. *Phanerochaete chrysosporium* a lignin-degrading fungus produces various oxidoreductive enzymes, including lignin peroxidase (LiP) and manganese peroxidase (MnP). Bacteria lignin and lignin-derived aromatic compounds are transformed into valuable products such as vanillin; monolignols; pyrogallol; lipids; furfural, cis,cis-muconate; pyruvate; lactate; succinate; polyhydroxyalkanoate, ferulate; pyridine-2,4-dicarboxylic acid; and pyridine-2,5-dicarboxylic acid [91]. However, lignin valorization using the microbial approach is still not feasible at an industrial scale, and many studies are necessary to understand the biochemical mechanisms.

Phenols are important precursors for a range of pharmaceuticals, herbicides, plastics, epoxy- and polyurethane resins, and various cosmetics. Organosolv depolymerization is considered the most feasible solution to increase yield in catechol derivatives for direct depolymerization processes resulting from the liquors. Catechol is a model compound for solid fuels, as is present in biomass tar and tobacco smoke, and its chemical structure is related to those of lignin and brown coal.

### 3.3. Vanillin

Vanillin is a mono-aromatic compound used as a food-flavoring agent in pharmaceuticals and in the fragrance industry. It is extracted from vanilla beans, but its consumption exceeds that of the vanilla resulting from this procedure. Therefore, synthetic methods have been developed. However, the need for alternatives to non-renewable raw materials used in these syntheses has encouraged research on the use of renewable feedstocks for vanillin production. The production of vanillin on an industrial scale occurs via the oxidative depolymerization of lignin derived from the pulp and paper industry and was commenced in 1937 by the Salvo Chemical Corporation. Lignin extracted by sulfite pulping was used, but this production ceased due to a decrease in the number of sulfite pulping facilities, and the kraft pulping process was implemented. Vanillin has increasingly been produced from the cheap petrochemical guaiacol, and its production now covers about 85% of the vanillin demand. Oxidative lignin depolymerization using oxygen as the oxidizing agent in alkaline media enables the selective production of aromatic aldehydes (i.e., vanillin and syringaldehyde), and their respective acids acetovanillone and acetosyringone as oxidation products in low yields have been produced [92]. The presence of the catalyst and solvent increased the cleavage of β-O-4 bonds, resulting in increased selectivity toward vanillin (32.3–36.2%). Kraft lignin undergoes sequential solvent fractionation using acetone aqueous mixtures. The obtained lignin fractions exhibit specific structural characteristics and low content of impurities [93]. Developing an efficient and environmentally friendly separation process is one of the most important tasks toward the industrial application of lignin-derived aromatics. The Norwegian company Borregaard has produced vanillin from lignin extracted by the lignosulfonate pulping process for more than 50 years together with other lignin-based high-performance chemicals. Stable and robust catalytic systems for oxidative lignin conversion should be developed.

### 3.4. Adipic Acid from Lignin

The conversion of lignin residue to adipic acid occurs via its base-catalyzed depolymerization to low-molecular-weight fragments, followed by microbial conversion of these to cis,cis-muconic acid by genetically engineered *Pseudomonas putida* separation, and purification of the muconic acid and catalytic upgrading to adipic acid are necessary as final steps [94].

### 3.5. Carbon Fibers

Lignin-derived carbon materials, such as carbon fibers, carbon mats, activated carbons, carbon films, and templated carbon, can be obtained. Lignin from a lignocellulosic biorefinery is an ideal potential precursor of carbon fibers (CFs). Its application in this direction offers some advantages such as abundant reserves, renewable and high carbon content, and low associated environmental impact, and could have a substantial impact if some important processing and quality hurdles can be overcome [95].

Heterogeneity and purity of lignin are critical challenges in carbon fiber production. The first step in lignin-derived carbon fiber production consists of lignin being processed into fibers. Lignin-based fibers are prepared by melt spinning, wet-spinning solvent-swollen gel, solution spinning, and electrospinning [96], followed by thermal stabilization and carbonization. Spinning temperature (100–230 °C) varies with lignin type, glass transition temperature, and melt viscosity. Nanofibers result from electrospinning [97]. Such nanofibers are used in batteries, supercapacitors, fuel cells, structural composites, and filtration devices. During thermal stabilization, dehydration, CO and CO_2_ elimination, additional crosslinking, and other reactions take place; thus, the oxygen, hydrogen, and nitrogen are eliminated by evolution of the HCN, H_2_O, CH_4_, O_2_, H_2_, CO, and NH_3_. Carbonization occurs at T ≈ 2000 °C in an inert atmosphere. By variation of the heating rate, it is possible to control the brittleness and the morphology of the carbon fibers. Lignin-based carbon fibers are composed of >98% graphene carbon in highly ordered structure, but their mechanical strength is low due to impurities and low molecular weight of lignin, as in the case of kraft lignin [98,99].

Lignin-based CF quality could be profitable only for lightweight vehicles and in association with biorefinery developments. To achieve this, improvements in lignin quality, such as chemical modification; increased purity; blending of various types of lignin or with polymers such as PET, PEO, PP, PLA, and phenolic resins; and addition of chlorinated PVC and diisocyanate, should be carried out. In this direction, there have been reported studies on phenolated hardwood, acetylated softwood kraft lignin, pyrolytic lignin, organosolv hardwood, etc. In Europe, Research Institutes of Sweden, in collaboration with other research organizations and industries, have made an important contribution with a proposal of a plant with 50,000 t capacity [100], while in the USA, Oak Ridge National Laboratory and Michigan State University has also made an important contribution in this field.

### 3.6. Biochar

Biochar has been produced from LCB and technically hydrolyzed lignin as a byproduct through pyrolysis (T = 500–700 °C) or hydrothermal carbonization (T = 180–300 °C). During heating at high temperatures, the functional groups are gradually lost, resulting in materials with polycyclic aromatic structures and a high condensation degree. The second step of the process is physical and/or chemical activation at high temperatures [101].

Biochar characteristics correspond to a solid biofuel with reduced H and O amounts, while N and S contents are below the detection limit. The biochar obtained at 600 and 700 °C has a good quality, and it behaves as a microporous adsorbent, being suitable for selective adsorption; primary, secondary, and tertiary wastewater treatment; as a catalyst; etc. [102].

## 4. New Developments in Lignin Use as Raw Material for Some Polymeric Materials

Borregaard (Norway) is the world’s leading producer of lignin-based biopolymers as green alternatives to synthetic polymers from wood derived from sustainably managed forests. These environmentally friendly products can be used in a range of industrial applications and markets. They are nontoxic with a documented, favorable environmental footprint and are based on a sustainable raw material and thus do not compete with food production [103,104].

The chemical and structural recalcitrance of lignin, especially technical lignin, limits processability and chemical reactivity. Deconstruction of lignin is one route to obtaining aromatic building blocks as, oligomeric fragments for the synthesis of new polymers. The resultant products contain several functional reactive alkenes, aldehydes, and phenolic residues that provide sites for chemical modifications to impart polymerizability. The crude lignin, lignin-derived oils, or depolymerized lignins of reduced molecular weights and improved reactivity have been used to produce lignin-based phenolic resins/adhesives, polyphenolic/polyurethane foams, and epoxy resins and others.

### 4.1. Phenolic Resins

The phenolic structure of lignin is exploited to obtain phenol–formaldehyde resins, which are mainly used as wood adhesives, but a pretreatment should be applied to increase its reactivity toward formaldehyde. The replacement ratio of phenol with lignin should be less than 50 wt%. Purified organosolv lignin and softwood lignin are recommended [105]. Lignin reactivity in condensation reactions can be increased by methyllation, phenolation as well as enzymatically (in the presence of laccase and oxidase) [106] and thermochemically by mild hydrogenolysis or pyrolysis [107]. Costs and non-recyclability of enzymes are limiting factors. A report published in 2018 indicated a TRL = 8 level for biobased phenolic resin production, with many patents registered in China.

### 4.2. Polyurethanes

In order to develop high-performance polyurethane (PUR) materials containing lignin, it was used not only as macromonomer to substitute petroleum-based polyols but also as blending filler for the PUR industry. Applied pretreatments involve both extraction of lignin fractions with various solvents and chemical modifications, e.g., depolymerization/deconstruction/liquefaction, hydroxyalkylation, dealkylation, and esterification, which are performed to obtain a more reactive lignin suitable for synthesis of PUR products. Lignin/PUR blends were also prepared to enhance properties of PUR. A higher bio-substitution (20–25 wt %) lignin ratio demonstrated the potential industrial application of lignin for high-value-added sustainable PUR materials [108] such as PUR foams containing lignin, which are already on the market. Developments required in the use of lignin in PUR production and materials are depolymerization and fractionation of lignin into well-defined oligomonomers, avoiding chemical modifications, improved dissolution of lignin into polyols, and modulation of the properties of flexible PUR foams obtained by green synthesis from liquefied lignin, which should be achieved using the most efficient chain extenders. Applications have been found for PUR foams in packaging of furniture and for the interior parts of car seats [109]. Increasing lignin reactivity via its prefunctionalization with isocyanates is sometimes necessary [110]. Researchers and the industry should pay attention to life-cycle assessment (LCA) studies and technical–economic assessments [111].

Lettner et al. [112] studied the use of kraft lignin in phenol–formaldehyde (PF) resins for wood-based panels and in PUR foams. The importance of reactivity and constant quality of lignin as a raw material was evidenced.

### 4.3. Epoxy Thermosets

Different procedures have been used to obtain high-performance epoxy thermosets from biomass. The depolymerization products of lignins resulting from the mixtures of both softwood and hardwood were subjected to Dakin oxidation in order to increase their phenolic functionality, and then they were glycidylated to obtain mixtures of epoxy monomers. Biobased epoxy thermosets were conveniently prepared from these epoxy monomer mixtures. They displayed outstanding thermomechanical properties, and, at the same time, environmentally damaging purification steps are avoided [113]. In other studies, oxirane moieties were introduced to the refined fractions, resulting in lignin epoxides that were crosslinked with polyether diamines (Mn = 2000 and 400) to obtain lignin-based epoxy resins [114]. Liu et al. [115] prepared series of high-performance epoxy thermosets via the reaction of epichlorohydrin with lignin oligomers blended with renewable epoxied cardanol glycidyl ether and then cured with methyltetrahydrophthalic anhydride.

In conclusion, at present, kraft lignin and other types of lignin resulting from biorefineries with unique structures and properties can be considered as raw materials for at least eight types of products, three with polymeric structures, namely carbon fibers, polyurethane foams, and phenol–formaldehyde resins, and five, chemicals, specifically vanillin, aromatic monomers, phenols, eugenol, adipic acid, and also fuels.

## 5. New Developments in the Use of Lignin as a Bioactive Compound

### 5.1. Antioxidant, Antimicrobial, Antifungal, Antiviral, Antitumoral, and Drug Carrier Activities

Because of their particular characteristics, lignins are used in pharmaceutic, medical, and cosmetics applications as bioactive agents. The medical or therapeutic potential of lignin-derived materials, such as antimicrobial, antiviral, and antitumor compounds, in controlled drug delivery [116], tissue engineering, and gene therapy has been studied [116,117], and the effect of the lignin type on the properties of the obtained materials was demonstrated [118,119,120].

Lignin obtained from agricultural/forestry residues or paper-pulping wastewater is rich in aromatic structures and a potential natural antioxidant. The lignin structure offers different functional groups along a phenol ether backbone that render it predisposed for: (i) functionalization with surface-active groups; (ii) confer to them an amphiphilic character; (iii) phenolic groups that allow them to undergo interactions such as π–anion and π–hydrogen/hydroxyl; (iv) phenolic groups that are susceptible to oxidation; therefore, lignins act as natural antioxidants [121,122,123]. Lignins exhibit comparable/higher radical scavenging ability compared to synthesized commercial antioxidants [124,125], being promising renewable alternatives. Preparation of lignin nanoparticles and of the chemically modified compounds are efficient procedures to improve the antioxidant activity, due to the increase in the free phenolic hydroxyl content and to the decreasing bond dissociation enthalpy. The reaction mechanism explaining antioxidant activity is based on SET and HAT reactions occurring together; usually. The HAT reaction is quite rapid (seconds to minutes), while SET reaction takes longer. The phenolic hydroxyl content and the mechanical properties of lignin-containing composites are negatively correlated with variation of the molecular weight. Lignins as natural phenols offer interesting properties, such as enhanced mechanical, antibacterial, antioxidant, antiviral, and anti-inflammatory properties; UV-shielding activities; and steady biocompatibility. Studies on lignin-based composites in biomedical, cosmetic and cosmeceutical applications have been focused on their use in sunscreen, antiaging formulations, and as an excipient for the production of conventional tablets.

Antimicrobial properties of lignin must be explored more through extensive research to provide a replacement for the current, toxic antimicrobial products. There are several studies related to antimicrobial activity of lignin and materials containing it [126,127]. Both scavenging activity and antimicrobial activity are dependent on the biomass source and extraction procedure, showing the following trend: organosolv of softwood > kraft of softwood > organosolv of grass. The antibacterial performance depends on the type of lignin, the bacterial strain, and the testing conditions. Kraft lignin isolated from corn efficiently inactivated *Listeria monocytogenes* and *Staphylococcus aureus* but not Gram-negative bacteria or bacteriophages, while kraft lignin extracted from eucalyptus inactivated both Gram-positive bacteria, such as *Bacillus cereus*, *Staphylococcus aureus*, and *Pseudomonas aeruginosa*, and Gram-negative bacteria, such as *Escherichia coli* and *Salmonella enteritidis* [128]. Lignins and lignin-containing films showed high antimicrobial activities against Gram-positive and Gram-negative bacteria at 35 °C and at lower temperatures (0–7 °C). The antimicrobial activity of lignins also depends on the solvent polarity (as shown for ethanol, acetone, and DMSO). Storage led to an increase in the antimicrobial activity against *S. aureus* due to the degradation of lignin over time. Purification of kraft lignin has a negative effect on the antimicrobial activity, while storage has a positive effect. Lignin incorporated into complex systems, as in the case of lignin around silver nanoparticle (AgNP) cores, showed an excellent antibacterial performance against *Staphylococcus aureus* and *Escherichia coli*, while Ag^+^ had no environmentally adverse effects [129,130].

Antifungal activity also depends on lignin type and processing conditions. It has been found that antifungal activity against *Aspergillus Niger* was the best for kraft lignin extracted from eucalyptus, which exhibited better antifungal performance with respect to that of the spruce lignin extracted via organosolv [131]. Lignin–carbohydrate complexes extracted from biomass via different methods, such as acidolysis, fractionation, and enzymatic hydrolysis, efficiently inactivated the *Encephalomyocarditis* virus and herpes simplex virus (HSV) [132,133]. Lignosulfonate, with a structural similarity to heparan sulfate, has antiviral activity against HSV and human immunodeficiency virus (HIV) [134].

Porous structure of materials influences their geometry, density, high surface area, permeability, water solubility, and adsorption ability, features required for medical applications, especially for controlled drug delivery systems, wound dressing, and tissue engineering. Such characteristics are also possessed by many lignin-based/containing biomaterials [38,135]. The chemical features of the oligomers and polymers of monolignol C9-building blocks of lignins can be exploited in the pharmaceutical sector mainly as a material for matrices and carriers for drug delivery, such as acetylsalicylic acid or paracetamol [136]. Lignin, chemically derived carboxylated lignin and lignin-based materials increased the release efficiency compared to controls while protecting the active compounds [137]. However, some cases present challenges. As an example, the kraft process involves high temperatures and harsh chemicals, leading to irreversible damage to the reduction of ether bond linkages, mainly β-O-4 bonds, and highly condensed lignin. Therefore, some processed lignins and technical lignins.are less chemically reactive and more cytotoxic compared to native lignin In such cases, it is important to evaluate the biocompatibility of each type of lignin [138].

Lignins are able to encapsulate either hydrophobic [139] or hydrophilic drugs [140]. Lignin copolymers as multi-arm carriers with antioxidant lignin core and poly(glycidyl methacrylate-co-poly(ethylene glycol) methacrylate) derivative arms are highly efficient in gene delivery [141].

### 5.2. Lignin Chemical Modification

Lignin chemical modification generates a number of functional lignin-based polymers, which integrate both the intrinsic features of lignin and the additional properties of the grafted/modified polymers. Some techniques have been reported for lignin functionalization, such as methoxy group substitution, phenolation, demethylation, ring-opening, dehydration polymerization, condensation polymerization, crosslinking, solvolysis, and combinations thereof. Lignin derivatives and copolymers mainly deriving from chemical processes at hydroxyl groups present in their structure (including acetylation, esterification, hydroxymethylation alkylation, methylation, and phenolation arylation, epoxidation, etherification, and amination) via copolymerization, such as silylation and grafting, or physical methods (irradiation, freeze-drying, sorption, changes in surface properties of lignin by cold plasma, electron beams, etc.) are also utilized to induce enhancements in green polymer composites as advanced and sustainable materials because they offer better miscibility with other polymeric matrices, leading to improved performance of lignin/polymer composites.

Stimuli-responsive materials. pH-sensitive lignin-based materials have attracted great interest in various fields, such as biomass refining, pharmaceuticals, and detecting techniques. The pH-sensitive mechanism of the smart materials is dependent on the hydroxyl or carboxyl content in the lignin structure. A novel pH-sensitive material was obtained by incorporation of ester bonds between lignin and 8-hydroxyquinoline (8HQ). The pH sensitivity and the sustained amount of 8HQ released under alkaline conditions (pH = 8) was higher than that under acidic conditions (pH = 3 and 5) [142]. Alkali lignin, as an ionotropic crosslinker, has been used to obtain chitosan–alkali lignin thermo-responsive hydrogels [143] for wound-dressing applications.

### 5.3. Lignin Use in Agriculture

Lignin can be transformed into agrochemicals such as fertilizers, pesticides, liquid plastic films, feed, soil improver, and plant growth regulator [144] and can be utilized in the cultivation of edible fungi. Ammonia-oxidized lignin nitrogen fertilizer, lignin urea, and lignin sulfonate nitrogen fertilizer have the characteristics of slow nutrient release and leaching loss when irrigating crops are small. They can also reduce the number of required fertilization periods, which greatly reduces water pollution. Lignin is very attractive for the production of agrochemicals with improved efficiency in slowing or controlling nutrient/fertilizer release into the soil upon the biodegradation of lignin due to its biocompatibility, wide availability at low cost, and many reactive groups that allow the chemical binding of a wide number of nutrient-containing groups. The hydroxyl, carboxyl group, methyl oxygen base, and carbonyl active groups with others in the lignin structure can combine with a variety of heavy metal ions (ferrous ions, Zn^2+^, Ca^2+^, Mg^2+^, Cu^+1;+2^, etc.) and chelating micro-materials to in the preparation of controlled-release fertilizers [145,146]. Lignin from the pulp and paper industry or agricultural waste can be chelated to achieve microfertilizers with reduced cost and good biological reaction activity. Lignin-chelated micro-fertilizer has a slow release, low cost, good stability, and high biological activity.

Lignin-based slow/controlled-release fertilizers can be obtained using three main approaches, namely (i) chemical modification of lignin constituting the nutrient, (ii) use of lignin as a coating for the active ingredient, or (iii) application of lignin as a chelating agent for trace element release [145,147,148,149]. Nitrogen-containing groups attached to lignin via ammoxidation (a process for the production of nitriles using ammonia and oxygen) and Mannich reactions are used, but both methods use toxic chemicals. The main limitation of the coating is the uneven surface and presented cracks; therefore, the nutrient release is less controllable than in the products prepared via chemical modification. Therefore, there are needed improvements to optimize the coating process. In valorization processes, the production of lignin-derived humic substances is also an attractive direction. To convert lignin into humic-like materials, alkaline aerobic oxidation, alkaline oxidative digestion, and oxidative ammonolysis of lignin are applied. The obtained humic substances are useful in soil enrichment, fertilizers, wastewater treatment, water decontamination, and medicine [150,151].

The use of commonly known agrochemicals increases the agricultural productivity, but they have negative impacts on the environment. The administration of fertilizers and pesticides in cultivated areas is achieved by spraying of these compounds over large areas, but the changes in weather patterns and geographical shifts make it more difficult to cultivate crops in many regions of the world. More sustainable methods to manage fertilizers and pesticides in fields should be elaborated. The encapsulation of agrochemicals to develop more sustainable formulations, such as nanofertilizers, nanoherbicides, and nanopesticides, which provide the sustained release of active compounds, is needed. Nanoencapsulation reduces the rate of dissolution of the agrochemicals and allows their slow, sustained release [152,153,154,155]. For grapevines and other woody plants, sicknesses such as Esca sickness, caused by fungi, bacteria, and viruses, are a global challenge that increasingly leads to economic losses. Presently, there is no efficient fungicide with systemic action against Esca. Worldwide damage by Esca is estimated to be USD 1.5 billion and is expected to further increase in the coming years, due to climate change and the connected increasing stress on plants. Lignilabs GmbH in Mainz (Germany) has developed a platform technology that can encapsulate various substances into lignin nano- and microparticles. Polymer additives/3D printing technology has been used for treating Esca sickness. An aqueous suspension of lignin carriers (hollow nanospheres) filled with an adapted fungicide is directly injected into the plant, which allows for treatment of the sickness at the place of its origin. The product with the commercial name ESCApe acts instantaneously against Esca fungi, and it preventively acts on healthy vines to prevent the disease. ESCApe is active against a large spectrum of wood-destroying fungi and can potentially be used against pathogens on fruit trees, shrubs, ornamental plants, or in the forest. ESCApe suspension is injected in minimal dosage (0.8 mL) into a drill hole (Ø 6 mm, 35 mm long) oblique to the vine trunk. Once the injected product is distributed in the plant vessels, the lignin is degraded by the enzymes produced by the fungus, which liberates the pesticides. The fungus therefore destroys itself through its enzymes. In addition to the instantaneous treatment effect, the length of preventive action is 3 to 5 years. Due to lignin and its unique properties applied, the production and the application of ESCApe are completely sustainable as well as user- and environmentally friendly.

## 6. Lignin Nanoparticles

Cellulose and lignin of lignocellulosic biomass are widely explored for the formation of several nano-ranged particles [156]. The emerging applications of nano-based cellulose and lignins have been explored in different sectors, including the biomedical and environmental fields [157]. There are some difficulties in obtaining certain types of lignin nanoparticles (LNPs) because the lignin structure is of an aromatic material with multiple phenolic rings. Nowadays, these difficulties have been overcome, and many types of nanosized lignins are produced, such as irregular nanoparticles, nanospheres and hollow nanospheres, hollow nanotubes, and nanofibers.

The preparation of lignin-based nanoparticles (LNPs) transforms unordered and complicated lignin materials into ordered nanoparticles with uniform size and morphology and excellent properties such as controlled structures and sizes, better miscibility with polymers, and improved antioxidant activity. LNPs are obtained by different methods, such as self-assembly; solvent exchange; acid precipitation; polymerization; ultrasonication, such as from empty fruit bunches of oil palms [158,159]; crosslinking; electrospinning; and CO_2_ use as a non-solvent. In the self-assembly process, an ordered or organized morphology results because of some specific intermolecular noncovalent interactions such as hydrophobic, electrostatic, hydrogen-bonding, and Van der Waals interactions in the absence of any external factor. LNPs are obtained by solvent exchange following dissolution (in tetrahydrofuran, dioxane, dimethyl sulfoxide, acetone, ethanol changed by water); precipitation; ultrasonication; oil-in-water emulsions, which are used for microcapsules (0.3–1.4 μm); preparation by ultrastirring when lignin micro- and nanocapsules are obtained; or by electrospinning of softwood organosolv in the presence of 2 wt % FeCl_3_ [160,161]. Hollow lignin-based nanospheres of a diameter around 200 nm were produced. Firstly, lignin was dissolved in tetrahydrofuran (THF), and then water was added under very specific conditions. Such LNPs have been loaded with an anticancer drug, which was successfully released under controlled conditions. Lignin has no cytotoxicity and shows better biocompatibility than many other possible carriers. To fix the anticancer drug inside the lignin spheres, β-cyclodextrin was grafted on the lignin, prior to the formation of the nanospheres. Hydrolysis lignin, kraft/alkali lignin, lignosulfonate, enzymatic hydrolysis lignin, and organosolv lignin can be used for LNP preparation. The β-Cyclodextrin-modified LHNPs exhibited a good sustained-release capability for the antitumor hydroxycamptothecin (HCPT) drug [162]. Modified lignin has an improved network structure and increased specific surface area and porosity. The obtained hollow nanoparticles encapsulate and load the drugs via self-assembly. The irregular lignin-based particles are ordered into the colloidal spheres (r = 110 nm) by dissolving acetylated lignin in THF followed by gradual addition of water [163]. Various combinations of different types of lignin, such as enzymatic hydrolysis lignin, kraft/alkali lignin [164,165,166,167], lignosulfonate [168,169], and organosolv lignin [170,171], and chemical modification of lignin or solvent/non-solvent systems have been used by several authors [172,173,174]. Utilization of the hazardous and expensive chemical reagents, such as acetyl bromide, cyclohexane, dioxane, NaNO_2_, maleic anhydride, THF, and acetone or processes involving complicated chemical modification reactions should be avoided.

### 6.1. Green Approaches to Preparing LNPs and Their Properties

The traditional manufacturing techniques of LNP preparation are costly and often toxic and with a high impact on the environment. Ecological approaches, which are simple and safe, have been developed for the synthesis of LNPs [175]. Green approaches to preparing LNPs include non-solvent nanoprecipitation and ultrasonication [176]. A very simple approach for producing LNPs with wide suitability and high efficiency consists of their processing in aqueous sodium p-toluenesulfonate (pTsONa) solution (APS-LNPs) at different pHs, at room temperature, without any other modifications. This method was applied to various types of lignin [177]. The hydrodynamic diameter of the LNPs varied with lignin type and decreased with the increase in COOH content at an initial processing pH < 7. Their zeta potentials decreased from −22.6 mV to −35.2 mV. LNPs from various production techniques show a specific variation of the minimum cytotoxic concentration (MCC) with solution concentration. In cytotoxic testing of LNPs using the Cell-Counting Kit-8 (CCK-8) assay, standard mouse fibroblasts, namely NIH-3T3 cells, were used. The obtained cell viabilities were over 90% when the concentrations were less than 800, 500, and 200 μg/mL sodium p-toluenesulfonate (pTsONa) solution (APS-LNPs) and THF/ethanol/water–LNPs respectively. Therefore, LNPs prepared using APS are less cytotoxic than many other nanomaterials.

LNPs have been obtained with high yield; therefore, a scale-up production of this procedure is promising. The properties of the LNPs depend on the synergistic behavior of the entrapped pTsONa and the intrinsic phenolic OH and COOH groups of the LNPs. Drug-loaded LNPs can be simultaneously prepared. Three types of technical lignins, namely BLN birch lignin (hardwood, BB), alkali Protobind 1000 (grass, PB), and kraft LignoBoost (softwood, LB), have been compared in an LNP preparation study utilizing non/anti-solvent precipitation, using as the lignin solvent 70% aqueous ethanol, acetone/water (3:1) and NaOH and water/aqueous HCl as the non-solvent. The acetone/water (3:1) system was found to be the most effective because it allowed production of small, spherical, homogeneous, and monodisperse LNPs with a negative surface charge and smooth surfaces [178]. Another simple and sustainable synthesis approach to obtaining LNPs was developed using a recycled γ-valerolactone (GVL)/water binary solvent through nanoprecipitation (dropping or dialysis) of the lignin solution. The highest yield of LNPs reached 90%. Their diameter was ca. 250 nm, and their zeta potential was −40 mV. LNPs showed good dispersibility and stability in water. LNPs can be used to stabilize essential oils and to promote their growth inhibition activity against microbes; therefore, they exhibit enhanced antimicrobial activity [179].

### 6.2. Lignin Nanoparticle Applications

LNPs showed superior antibacterial and antioxidant/UV barrier properties. Lignin-based hollow nanoparticles are considered suitable for many applications both as additives in polymers and in therapies. There are various fields for industrial applications of LNPs, such as in drug delivery carriers, UV absorbents, hybrid nanocomposites, antioxidant and antibacterial agents, adsorbents for heavy metal ions and dyes, and anticorrosion agents’ nanofillers [180]. Entrapment, encapsulation, adsorption, and covalent binding are common methods for loading active compounds into lignin materials. LNPs as drug delivery nanosystems are capable of loading both hydrophobic and hydrophilic drugs for targeted cancer and tumor treatments [181], including poorly soluble curcumin, hydrophobic coumarin-6 [182,183], bioactive molecules of resveratrol, hexadecane, sorafenib (SFN), benzazulene, capecitabine [37,165,166], hydrophilic rhodamine 6G, and doxorubicin hydrochloride (DOX) [160,165]. LNPs form through the supramolecular assembly of poorly water-soluble molecules via electric interactions with aromatic rings. Most of the substances that have been successfully entrapped in lignin NPs to date are poorly water-soluble, low-molecular-weight compounds, such as antibodies and enzymes.

pH-responsive lignin-based nanocapsules have been obtained by interfacial miniemulsion polymerization. Sodium lignosulfonate grafting with allyl groups takes place via etherification. An oil-in-water miniemulsion system has been prepared through ultrasonication and dispersion and has been used for a thiolene radical reaction with lignin-based coumarin-6 [168]. Curcumin, a polyphenolic natural compound, can be delivered by LNPs obtained by modified phase separation [168]. Curcumin was used as a lipophilic drug model, as its poor solubility and low oral bioavailability limit its therapeutic efficacy. The average particle diameter of curcumin-loaded LNPs was 104 nm, and the encapsulation efficiency was 92%. They also showed enhanced protection of the entrapped curcumin under storage conditions and are also stable in simulated gastric fluid and slow release under intestinal conditions. In vivo pharmacokinetics tests demonstrated that the LNP system increased by ten-fold the bioavailability of curcumin in comparison to common administration. Temperature and pH at which the entrapped drug is released are other important parameters for drug delivery systems [171]. They are extensively studied, especially as drug and gene delivery systems and in cancer therapy [175,181,183].

The antioxidant and UV protection properties of nanosized lignin have been utilized in the food, pharmaceutical, and cosmetic industries [184,185] (Table 2). Two sets of nanocellulose-based cryogels that differ in their overall surface charge density have been prepared by freeze-drying, namely those containing anionic LNPs anchored to cationic cellulose nanofibrils (cCNFs) and or cationic LNPs (cLNPs) that are combined with anionic TEMPO-oxidized CNFs (TCNFs) [186]. LNPs acted as crosslinkers and affected rheological and water holding capacity as well as the firmness of the cryogels. The cryogels are pH dependent, regenerable, and reusable, being suitable for cationic, anionic, and neutral aromatic pharmaceuticals and adsorption of the anionic aromatic pharmaceutical diclofenac of the aromatic cationic metoprolol and tramadol and of neutral aromatic carbamazepine. As crosslinking agents, the LNPs or cLNPs affected the rheological behavior of the cryogels. Reusable adsorbents, such as macroporous cryogels resulting from anchoring lignin nanoparticles (LNPs) to the nanocellulose network via electrostatic attraction, have been developed. They are useful for simultaneously removing pharmaceuticals of varying chemical structure and properties from polluted media [186].

**Figure 3:**
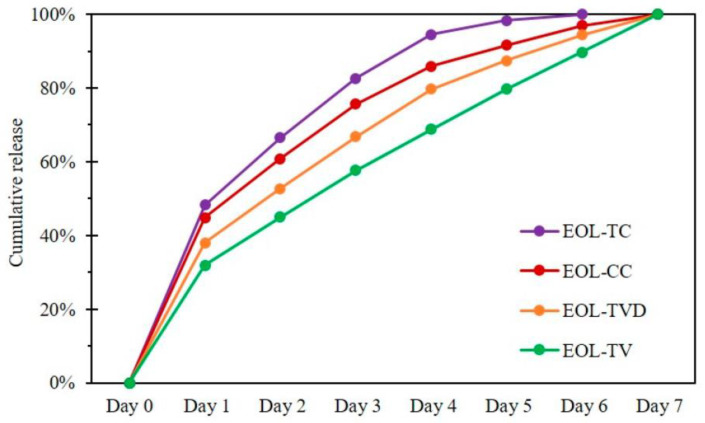
Cumulative release profiles of essential oils from LNPs containing C. *capitatus* (EOL-CC), T. *capitatus* (EOL-TC), T. *vulgaris Demeter* (EOL-TVD), and T. *vulgaris* (EOL-TV) oils (reprinted from [187]).

Essential-oil-loaded LNPs showed interesting antifungal activity against both white-rot fungi and brown-rot fungi [187]. Cumulative release profiles of EOs vary with the EO type and lignin concentration in loaded LNPs, as shown in Figure 3, while minimal inhibitory concentration (MIC) also depends on fungus type (*T. versicolor*, *G. trabeum*, *P. ostreatus*, and *P. monticola*), ranging from 0.05 mg/mL (*P. monticola*) up to 0.60 mg/mL (*P. ostreatus*).

Antibacterial and adhesive hydrogels, such as core-shell nanoparticles and Ag-LNPs, embedded into pectin/acrylic acid polymeric networks have been obtained [188].

Softwood lignin nanoparticles (SLNs) are promising alternative bio-additives for enhancing PLLA crystallizability toward the development of fully biobased and biodegradable plastics [189].

Lignin macromolecules have been in situ modified by mild ozone oxidation without significant degradation of carbohydrate polymers (i.e., cellulose and hemicellulose) in lignocellulose nanofibrils [161]. The interfacial hydrogen-bond energy was improved, and, by molecular dynamics simulation, the deformation process of lignocellulose nanopaper was validated. Lignin acts as a functional component in the lignocellulose nanopaper matrix, but the interfacial hydrogen-bonding among lignocellulose nanofibrils is diminished, and mechanical performance is decreased. Ozone oxidation of 40 min leads to changes in the carboxyl content onto nanofibers, so that the zeta potential values, the crystallinity index, and the degree of polymerization were reduced, while the lignocellulosic structures were preserved. The kappa number decreased from 78.9 to 68.3, while the whiteness of the lignocellulose nanopaper increased from 15.2 to 38.8. The ozone preferentially oxidized chromophoric structures of lignin. Lignin-modified lignocellulose nanopaper preserved its water and thermal stability, mechanical properties, and optical performance. The developed lignocellulose nanopaper is a multifunctional and flexible material used in electronic applications. Multifunctional ozone oxidation substrate was printable and highly compatible with conductive materials (as silver), providing an eco-friendly alternative to conventional substrate materials (e.g., Mylar film and polyimide).

### 6.3. Lignin Nanofibers

Nanofibrous networks that closely resemble the native extracellular matrix (ECM) for new drug delivery systems and other biomedical applications have been developed by electrospinning [190]. They offer a high surface area, high porosity, pore interconnectivity, resistance to agglomeration, and high drug loading and encapsulation capacity, etc., properties that make them excellent candidates for drug delivery. A wide variety of bioactive compounds or therapeutic agents can be incorporated within the fibrous meshes [191]. Heterogeneous chemical composition, complex branched structure, and low molecular weight are challenges to electrospinning pure lignin. Pure lignin nanofibers were obtained using a coaxial electrospinning technique via the optimization of the electrospinning parameters or using suitable solvents [160,192]. The spinnability of lignin is improved by its combination with various hydrophilic or hydrophobic polymers, such as PLA [193], polycaprolactone (PCL) [194], polyvinyl acetate (PVA) [195], and polyhydroxybutyrate (PHB) [196].

Abudula et al. used coaxial electrospinning to encapsulate chitin/lignin gels with polycaprolactone (PCL) for fibrous scaffolds with controlled drug release ability [197]. The minimum fiber diameter, porosity, biodegradation, optimum pore size, tensile strength, and Young’s modulus were improved by lignin (10 wt%) addition.

Antioxidant PLA/lignin nanofibrous scaffolds have been applied for cartilage tissue engineering and osteoarthritis treatment. It has also been suggested that the scaffolds can be used in many other biomedical applications, including delivery of various bioactive molecules, UV filtration, and consumer care [198].

**Table 2:** Lignin nanoparticles (LNs) and electrospun-based lignin biomaterial properties and applications (recent studies).

LNPs and Other Components	Properties and Applications	Refs
Lignin nanoparticles (LNPs)/starch	Green nanofiller in starch-based biocomposites; strong interfacial hydrogen bonding; enhanced mechanical properties, thermal stability, antioxidant activity, and good (UV) irradiation shielding performance; applications such as delayed oxidative deterioration of soybean oil/advanced food packaging	[199]
Lignin nanoparticles obtained by a acidolysis process from corn lignin/polyurethane, polyethylene glycol and diisocyanates	LNPs as crosslinking agent in polyurethane nanocomposites. Composites with hydrophobic, UV resistant and dielectric properties, enhanced tensile strength; good reprocessability	[200]
0.5 wt% lignin and nanolignin in poly(lactic acid) composites synthesized by in situ reactive processing as melt mixing and ring-opening polymerization (ROP)	Reactive processing produced nanolignin-containing composites with superior crystallization, mechanical, and antioxidant properties. LNPs act as a macroinitiator in the ROP of lactide, resulting in PLA-grafted nanolignin particles, improved dispersion and the formation of interfacial covalent bonds facilitated by LNPs	[201]
Lignin-based nano-micelles using the “grafting-from” method	pH-sensitive, biocompatible, suitable for oral drug delivery	[202]
Nanolignin/chitin nanofibrils and complexes loaded with active molecules, such as vitamin E, sodium ascorbyl phosphate, lutein, nicotinamide, and glycyrrhetinic acid	Spray-drying method; nanostructured chitin; Functional agents in skin regeneration and antimicrobial, anti-inflammatory, and antioxidant activities; cytocompatibility in skin regeneration.	[203]
PCL coated with chitin–lignin gel; shell/core fiber, coaxial electrospinning; electrospun PCL/lignin, initial blending	Methylene blue, penicillin/streptomycin; sustainable drug release; wound dressing; fibrous scaffolds; MTT tissue engineering	[197,204]
LNP/PVA/PVP electrospinning. Initial blending	Paclitaxel local anticancer therapy	[205]
AgNP-loaded electrospun PVA/lignin nanofibers	Membrane filtration, antimicrobial fabrics, and wound dressing	[206]
Lignin/cellulose acetate/N-vanillidene-phenylthiazole copper (Il) complexes, nanofibrous materials	Antimicrobial for hygienic applications	[207]

Electrospinning is generally used to fabricate 2D dense fibrous scaffolds with limited cell infiltration. By coupling electrospinning with other advanced manufacturing techniques (e.g., 3D printing and cryogelation), the nanofibers will be more efficient for drug delivery applications [208,209]. Lignin and LNPs are biodegradable and offer good performance and special properties, but their utilization in the medical fields is still very low. It is expected that the surface modifications with various functional groups will increase their application in medicine, especially in drug delivery. The development of alternative preparation methods for LNPs, nanocomposites, and hybrid materials with low cost, biocompatibility, and sustainable surface modification, that are eco-friendly and easier for large-scale production, is necessary, especially for medical applications.

Developments in lignin valorization also involve important applications, such as lignin-based hydrogels, three-dimensional printing materials, electrospun scaffolds, surfactants, electrodes, and production of fine chemicals.

## 7. Lignin-Based Hydrogels

A drastic increase in the number of published articles, 336 (1990–2022) in the field of lignin hydrogels, suggests the importance of this research area [38,210] or future development, with 55 publications on lignin in drug delivery in 2022 and with most of them being focused on LNPs [211]. A major prerequisite for lignin with respect to synthesizing hydrogels is the abundance of phenolic OH groups in the lignin backbone which can crosslink with COOH groups of the co-joining monomer. The highest concentration of phenolic OH groups is found in kraft lignin, followed by organosolv and soda lignin. In terms of mechanical properties, soda lignin-based hydrogel is superior, since strong crosslinks will result in improved elastic nature. Organosolv-based hydrogel may be the best choice, while superior mechanical properties will necessitate the use of soda lignin-based hydrogels.

The three-dimensional network of hydrogels can be easily adjusted by the degree of crosslinking to achieve the desired physical properties, elastic modulus, and gelation time suitable for in situ use degradation rate, so they can act as indicators. The increase in the crosslinking degree leads to inferior water swelling capability. Thus, shape and flexibility can conform to the target tissue, and the degradation period can be tuned to follow the progress of tissue regeneration [212]. The biodegradability of the natural polymers and their non-inflammatory effect and nontoxicity of these degradation products suggest that these types of materials are suitable for being used in tissue regeneration. Nanogels obtained from natural polymers have become very important in many scientific and industrial domains, due to a series of interesting properties, such as their large surface area, significant swelling, high ability for loading active substances, and flexibility. The design and the production of nontoxic, biocompatible, and biodegradable micro/nanocarriers, demonstrated their suitability and feasibility for biomedical applications, such as drug delivery, tissue engineering, and bioimaging [213,214]. Controlled hydrogels or highly functional hydrogels are used in drug or bioactive substance delivery systems as well as other clinical applications.

Due to its well-known properties (i.e., biodegradability, biocompatibility, low-toxicity, antioxidant, and antimicrobial properties), lignin is often used as an important component of hydrogels. Furthermore, lignin-containing hydrogels are more environmentally friendly when compared to synthetic hydrogels. Because the high diversity of their functional groups, lignin-based hydrogels can be loaded and can deliver a large number of both hydrophilic and hydrophobic therapeutic agents, such as small molecules, nanoparticles, proteins, enzymes, and nucleic acids.

Different innovative physical, chemical, and biological processes have been elaborated to obtain lignin-based hydrogels [215].

Physical hydrogels are formed by intermolecular interactions, including self-assembly through hydrogen bonds and electrostatic, hydrophobic, and van der Waals interactions. Lignin-based macroporous hydrogels are obtained using either salt leaching or gas foaming methods, but from these processes byproducts with high toxicity can result, and the synthesis is a multi-step procedure with a high cost. Cryogels show major advantages over hydrogels prepared by classical methods because they have a highly interconnected pore network structure coupled with macro-sized pores [216]. The walls of the cryogels can be highly crosslinked and dense, and they act as depots for drug storage and improve mechanical properties, suggesting their potential use as scaffolds and in sustaining injectability and shape memory. Two distinct cryogelation procedures may be employed: (i) the freeze-drying approach with multiple freeze-drying cycles using traditional lignin hydrogel precursors, where each freezing cycle generates additional hydrogen/electrostatic bonds and strengthens the polymer network, and (ii) the freeze-thawing approach occurring in a frozen solvent via a single-step cryopolymerization step resulting in the polymer network [217]. Frozen aqueous solvent, such as porogen, induces the appearance of large pores following ice thawing [218]. The properties of products obtained by cryogelation, such as the size and the shape of the pores, their interconnectivity, and also the ability of drug adsorption, vary with lignin sources along with possible modification, temperature, and polymer concentration [219].

The polar sites on the lignin backbone can be involved in physical crosslinking with hydrophilic polymers by H-bonding (such as polyurethanes and lignin–chitosan–polyvinyl alcohol (PVA) composite hydrogel) [135,220]. Such hydrogels are obtained using several methods, such as heating in an oven for short periods of time when the crosslinking is hypothesized to be uniform, microwave irradiation, use of ultrasound, and reactive extrusion. All these techniques can be considered as green because they use no or few toxic solvents [221,222].

Chemical hydrogels are synthesized using crosslinking agents. Covalent crosslinking includes the use of carbodiimide compounds (e.g., 1-ethyl-3-(3-dimethylaminopropyl) carbodiimide/N-hydroxysuccinimide (EDC/NHS), and glutaraldehyde (GA)), radical polymerization, thermal gelation, enzymatic reactions, click chemistry, photo-oxidation, and radiation crosslinking, reversible addition–fragmentation chain transfer polymerization, catechol redox chemistry, crosslinking copolymerization, ring-opening polymerization, etc.

Semi-interpenetrating/interpenetrating networks result from polymerization techniques. Biologically mediated processes occur in the presence of laccases/peroxidases or a whole cell (fungal)-mediated process. The crosslinking grafted lignin procedure uses unsaturated monomers or other functional chemicals to improve its reactivity. Esterification of the phenolic hydroxyl group of lignin resulted in unsaturated grafted lignin that was copolymerized with hydroxyethyl acrylate, resulting in lignin-based hydrogels. The “graft-from strategy” leads to the production of polymers starting from the active sites into the polymer backbone, while the “graft-onto” procedure combines the synthetic polymers with lignin (as the result of creating covalent links between the lignin backbone and the terminal groups of the graft polymers). Both strategies are based on the two procedures used to obtain hydrogels, namely atom transfer radical polymerization (ATRP) and fragmentation chain transfer (RAFT) polymerizations. The obtained networks of interpenetrating lignin/polymer such as hydrogels have the perfect aligned structures and controlled properties [215,223,224].

Compared with the microbial attack point of view, hydrogels with a high crosslinking degree proved to be more resistant than hydrogels with low crosslinking, due to the reduced accessibility of ligninolytic fungi and actinomycetes [225]. It is known that the most ligneous fungi have enzyme systems that attack the phenolic substructures in a direct way; therefore, lowering the hydrogel concentration can protect them from a fungal attack. Controlled biodegradation is still a target with high importance in drug delivery domain [226].

Due to population growth, the use of pesticides has significantly increased, as along with the demand for agricultural products. In the meantime, waters have been also polluted with pesticides, resulting in serious negative effects on human health and on the environment. In order to remove pesticides from the aqueous environment, activated carbons or biobased materials represent important alternatives due to their properties (i.e., bioactivity, biodegradability, and biocompatibility). Sustainable solutions to remove pesticides from aqueous environment consist of using activated carbons or biobased materials due to their complementary functionalities such as bioactivity, biocompatibility, biodegradability, and unique chemistry [210,227,228,229]. Aerogels, xerogels, and cryogels as mesoporous materials with low density, high porosity, and tunable surface chemistry can be useful for environmental remediation applications. For example, aerogels remove CO_2_ and volatile organic compounds from air as well as ions of heavy metals, oils, and organic pollutants from water.

Applications have been found for lignin hydrogels in agriculture (sustained/controlled water absorption, slow release of pesticides/fertilizers), being superior in terms of mechanical properties (such as tensile strength and compression resistance), environment protection (heavy metal adsorption, dye removal, water treatment), suitability for food packaging and the biomedical sector [230] (wound healing/dressing, anti-microbial coatings on medical implants, oxygen scavenging, tissue engineering, sustained drug release), and preventing spontaneous combustion of coal, biosensors, and electronics (supercapacitors) (Table 3).

Lignin hydrogels are stimuli-responsive to pH, temperature, ionic strength, etc., being desirable for the development of drug delivery systems [20,215]. Hydrogels with a significant content of lignin are characterized by a high cumulative drug release because they exhibit an improved viscoelastic behavior, adequate structure of pores, and good swellability. Between their significant disadvantages, one can mention their low mechanical properties, fast drug release kinetics, and non-adherent nature (a supplementary dressing being necessary in order to protect them), and it is not always easy to obtain hydrogels of a pure or high lignin content due to the polydispersity and differences in the molecular structure of lignin [230].

Antimicrobial, antioxidant, and anti-inflammatory intrinsic properties of lignin make it a valuable therapeutic agent, and it is beneficial in other medical applications. It enhances the viability, propagation, and the differentiation of Schwann cells and, ultimately, facilitates nerve tissue generation. Antimicrobial activity is explained by the interaction of the hydroxyl group of phenolic compound lignin with the bacterial cell, causing cell membrane disruption, the infiltration of cell components, and ultimately bacterial cell lysis. The antioxidant ability of lignin also has proven to be useful in composite hydrogels for biomedical applications [215] that still are in the lab research stage. However, their commercialization continues to present various difficulties, such as lignin isolation, standardized molecular weight, lignin structural characteristics, and lignin cost. Functionalization of lignin to improve its polymerization with other polymeric materials is an open direction for research, and in vitro and in vivo detailed toxicity studies are required to facilitate the application of lignin-based hydrogels in the food and pharmaceutical industries. Cost-effective technology, scaling, and proof of concept (POC) to attract industrial production are required for lignin-based hydrogel commercialization.

*Other applications*: Composites of bio-polyamides reinforced with lignin, which also acts as a fire retardant, have been obtained [259]. Sodium lignosulfonate in polypropylene matrix is a surfactant, flame retardant and smoke suppression agent [260]. Practically, lignosulfonate (with a structure of hydrophobic aromatic rings and hydrophilic sulfonate groups) biosurfactants have a wide range of applications, including as a substitute for standard surfactants, as an asphalt emulsifying and wetting agent [261], and solubilization and stabilization of thymol in water [262]. Sulfethylated lignin (similarly, butyric anhydride-modified kraft lignin and PEGylated lignin) can be a surfactant, stabilizer for oil–water emulsions, reinforcement, UV protection additive, and antioxidant, and surfactant activity offers a green and economic route to obtaining strong and transparent polymeric nanocomposites containing ~30 wt% LNPs [263] as well as a *stabilizer/surfactant* for oil–water emulsions [264]. Nanosized lignin using soda lignin from empty fruit bunches of oil palms is nontoxic and superior to the commercial emulsifying agent, and sulfethylated lignin was established to be a good emulsifier for water-in-oil emulsions.

## 8. Three-Dimensional-Printing-Based Lignin Biomaterials

“Additive manufacturing (AM) or 3D printing is a digital manufacturing computer-aided design process to fabricate layer-by-layer products with customized sizes, shapes, and functionality” [265]. It is performed for medical/pharmaceutical or food purposes using different techniques, such as stereolithography (SLA), digital light processing (DLP), direct ink writing (DIW), fused deposition modeling (FDM), selective laser sintering, selective laser melting, 4D/5D/6D printing, melt electrowriting (MEW), and cryoprinting [266,267,268,269,270]. AM is utilized to address the demand for production of pharmaceuticals with patient-specific dosages [271], customized drug formulations [272], the most desirable geometrics [273], and controllable drug release profiles. AM should be available in hospitals or medical care centers for printing tailored personalized medicine or delivering therapeutic agents [274]. Lignin-based materials meet both printing and medical requirements, such as appropriate printability, mechanical properties, biodegradability, biocompatibility, tissue biomimicry, and non-cytotoxicity [275]. They have been used to design meshes for wound dressing, capsules for pharmaceutical oral drug carriers, and innovative hydrogel bioinks for 3D bioprinting in tissue engineering, microextrusion, and laser-assisted bioprinting techniques [276,277].

AM techniques, such as extrusion printing, direct ink writing, and fused deposition modeling, are suitable for printing pure lignin, lignin derivatives, or a combination with other polymers or monomers as fillers or additives necessary to create lignin-based printable feedstocks [278,279]. For printing lignin-based biomaterials, extrusion and vat photopolymerization are the most commonly used technologies [280,281,282]. Lignin-coated PLA composite pellets containing drugs printed by fused deposition modeling into various mesh sizes have been obtained [283]. Antimicrobial and antioxidant filaments composed of lignin and polybutylene succinate (PBS) have been studied by Abdullah et al. [267,284], using printing based on fused deposition modeling and coated with silver/zinc oxide (Ag/ZnO), which exhibits a strong inhibitory effect against bacteria to fungi. A Ag-LNP nanocomposite inserted in a methacrylated O-acetyl-galactoglucomannan (GGMMA) network is a good printed antimicrobial hydrogel with strong bactericidal performance [285]. Domínguez-Robles et al. [286] designed a bioactive wound dressing by loading curcumin (an anti-inflammatory and antimicrobial agent) and D-Panthenol onto PCL/lignin for 3D printing. The printed composite displayed sustainable release of bioactives and antioxidant, antimicrobial, and anti-inflammatory properties.

The large-scale utilization of lignin in 3D printing is still a great challenge due to its inherent brittleness and non-thermoplasticity. Thermoplastic polyurethane regulates the rheological properties of lignin for 3D printing [287]. Other examples are given in Table 4.

Lignin is physically or chemically mixed with other components, such as cellulose or its derivatives, to take advantages of each component to formulate high-performance 3D bio-feedstocks for 3D products. Several lignin-based compositions of inks used in 3D printing are known as lignin/poly(ethylene oxide) (PEO), 20–40% lignin/acrylonitrile butadiene rubber (NBR41)/acrylonitrile-butadiene-styrene (ABS);/polyacrylonitrile (PAN)-based carbon fiber (CF); lignin modified with nylon 12/acrylonitrile butadiene rubber (NBR41) [275]. Lignin/poly(ethylene oxide) (PEO) blend filaments for 3D printing with a melt-spinning process have been prepared. The filament derived from lignin with 15% PEO had a uniform diameter, smooth surface, adequate mechanical properties, and thermal stability. Bio-feedstocks in the presence of lignin allowed good printability at lower temperatures. Many existing processes operate at very high temperatures, being expensive and difficult to scale. To address these challenges, researchers from the University of Delaware replaced methanol, a traditional solvent used in lignin deconstruction, with glycerin to enable the process to take place at a normal atmospheric pressure [296]. Replacing methanol with glycerin provided the same chemical functionality but at a much lower vapor pressure, leading to a more cost-effective system.

## 9. Lignin Use in Green Functional/Complex Materials

Green polymer biocomposites generally consist of a biobased thermoplastic or thermoset matrix with organic (e.g., wood flour, chicken feather) fillers (particulates or fibers) and/or organic matrices (biopolymers) with inorganic additives such as carbon black, clays (montlorillonites, haloysite, glass fibers), or silver nanoparticles (AgNPs).

The possibility of creating lignin-based polymer biocomposites to replace petroleum-based composites has attracted the interest of many researchers worldwide due to the negative environmental impact of traditional composites over time. Their aromatic and crosslinkable functional groups make them suitable for a variety of polymeric matrices with improved wettability, strength of the polymer composites, mechanical properties, and new properties such as fire-retardant and UV-light-blocking characteristics [297,298]. Lignin, with its distinctive chemical structure, can function in lignin-based biomaterials including biocomposites, copolymers, nano-/micro particles, fibers as a stabilizer, compatibilizers, and flame retardants. Lignin’s role is very different as an eco-friendly biodegradable and renewable matrix, crosslinking agent, and nano- and micro-filler, being used in building the designed structure and in modifying some properties such as mechanical, thermal, antioxidant, antibacterial, biodegradability, biocompatibility, permeability, porosity, water solubility, and adsorption ability. However, it can only be incorporated in small amounts in other polymers because of its rather poor miscibility.

Stabilized morphology and improved thermal properties with lignin as an additive increased the number of phenolic groups, ketone molecules, and chromophores, this leading to higher UV light barrier properties, as lignin exhibits a strong UV-blocking capacity, as well as antioxidant and radical scavenging characteristics. Lignin has been investigated as an active ingredient to create film materials with reduced hydrophilicity and ultraviolet-blocking qualities.

The differences in characteristics between lignin and conventional polymers led to the incompatibility of the biocomposite. Its highly hydrogen-bonded network through phenolic groups makes it immiscible with many polymers. Solvent casting and extrusion methods are mainly used to incorporate lignin into polymeric matrices. Chemical modification, which depends on lignin origin and on the extraction conditions, was applied to obtain better compatibility of lignin with polymer matrices in bi- and multiphase composites [299,300,301,302]. Deconstruction and chemical modifications will increase lignin utilization in complex materials. Modified lignins are useful materials for environmentally friendly polymeric materials. However, chemical modifications require additional investments in the form of solvents, reagents, and energy inputs.

After cellulose, lignin is the most commonly used natural polymer in green and synthetic biomaterials. Introducing lignin into commercial polymers can create green functional/complex materials such as composites or eco-friendly composites, with very interesting properties. Lignin contributes to improving properties, such as dimensional stability, hydrophobicity, porosity, material density, mechanical properties, biodegradability, biocompatibility and non-cytotoxicity; lignin can also possibly impart functionalities of the resulting products, such as antibacterial and antioxidant properties and controlled drug release, at lower production costs. LNPs are also used. Using strategies inspired by nature, different researchers have developed lignin-based nanocomposites that display both the well-known properties of bio/nanocomposites (improved mechanical and gas barrier properties and higher thermal stability) and the remarkable features of either the biological or inorganic (sometimes nanoparticles) moieties, such as biocompatibility, biodegradability, and, in some cases, functional properties. Lignin can be incorporated into the biocomposites as a nucleating agent, UV absorber [45,303], copolymer, filler material, reinforcement, coupling agent, compatibilizer, etc. Lignin-based biocomposites are applied in drug delivery systems, wound dressing, tissue engineering, and regenerative medicine (e.g., skin, nerve, cartilage, bone, and hard tissues). However, the valorization of lignin for high-value applications in composite materials is low, which could result in large increases in costs. Its use as a reinforcement material in biopolymers is a comparatively new and emerging research field [304].

Although lignin is highly resistant to chemical and biological degradation, it is slowly degraded by ligninolytic microbes [305]. Lignin has been used as the main matrix in a new class of engineering materials based only on renewable resources. For example, the Arboform^®^ composites consist of isolated lignin, natural fibers, and natural additives, while Xylomer™ (made up of lignin/polymer blends) materials are elaborated through conventional thermoplastic processes, such as injection molding [306]. Lignin use in composites manufacturing is limited by its low solubility and reactivity, high polydispersity, glass-transition temperature (Tg), sulfur content, color, etc. Selection of a suitable lignin type and its deconstruction methods, functionalization/modification, and the incorporation of UV stabilizers can be applied [307,308].

Carbonyl and conjugated phenol groups, such as chromophores, inside the lignin structure explain the light absorption in the UV range (200–400 nm), thus showing very good UV shielding and being a promising material to substitute the synthetic UV absorbers in composites, mainly in food packaging, because they help to protect food from UV irradiation. The chromophore groups also impart an undesirable dark color, creating a loss of visible transparency of the polymer film. Visible transparency is an important factor for food packaging because customers generally desire to see the product inside the packaging. The UV-absorbing lignin composite materials can be included in food packaging, healthcare products, and solar panel protection [36]. The UV-shielding properties and color of lignin are modified by selected extraction process, chemical modification method, and size of lignin particles, etc. The synergetic effect between lignin and the other constituents of the complex materials was demonstrated. More efficient lignin dispersion and compatibilization will lead to improvement in the properties of the multicomponent materials.

As mentioned before, other types of materials (LNPs, hydrogels, etc.) have many possible applications in agriculture, biomedicine, and the food and building industries, and different lignin-containing materials such as LNPs as well as 3D-printing can help to reduce the quantity of waste products and decrease environmental pollution [308].

Possible ways to improve the dispersibility and compatibility are lignin esterification [309,310,311], the use of crosslinkers [312,313,314], and polymer surface modification using physical or chemical methods [315,316,317,318].

**Figure 4:**
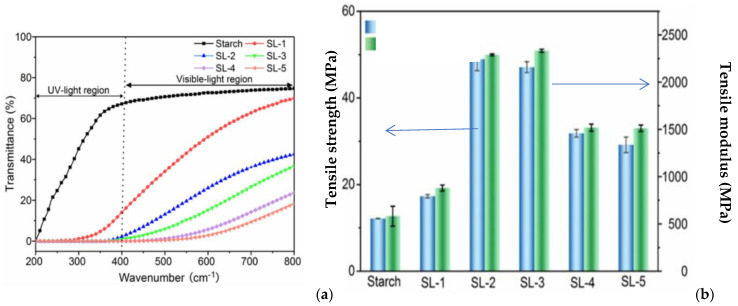
UV–Vis light transmittance spectra (**a**) and mechanical properties of starch film and starch/lignin composite films containing: 1% LNPs (SL-1); 2% LNP (SL-2); 3% LNP (SL-3), 4% LNP (SL-4 and 5% LNPs (SL-5), respectively **[324]**.

The most useful blends/composites containing lignin are those biobased polymers (such as starch, cellulose, epoxy, natural rubber, chitosan, alginate, plant proteins) and synthetic ones (polypropylene (PP), PVA, polylactic acid (PLA), polyhydroxy butyrate (PHB), poly(ethylene glycol) (PEG), poly(vinylpyrrolidone, polyamides, etc.). There is a special interest in obtaining and improving the green composites containing lignin (Table 5). Corn was blended with soda lignin and graphene oxide in a wheat starch matrix. Different combinations have been tested such as corn starch, cassava starch, urea-crosslinked tapioca starch, kraft and acid hydrolyzed lignin in modified corn starch microparticles with crosslinking agents, adipic acid in corn starch and glycerol, and lignin–sago starch blends for novel food packaging film using a solvent casting process. The films with kraft and soda lignin as fillers have an improved water barrier and thermomechanical and seal strength properties and a considerable reduction in permeability (water vapor) and transmission, particularly when films are made from hydrophilic materials, such as alginate and starch, because of the interaction of lignin and the hydrophilic groups of the biopolymer [319,320,321]. Starch/20% kraft lignin foams obtained by compression molding showed similar properties to PS. Starch foams are biodegradable alternatives to foamed polystyrene [322]. Lignin incorporation reduces the oxygen and water vapor permeability. Lignin from the lignocellulosic ethanol process is further valorized for sustainability reasons. The obtained LNPs with an average diameter of less than 160 nm were used as reinforcement for green biodegradable starch films. The 20% incorporated lignin improved mechanical properties and stability of the composites. The presence of lignin-rich solid particles with high hydroxyl group content enhanced the hydrophilicity and the surface energy of starch/lignin film [323]. Lignin-starch films exhibit advantageous physical properties, such as low odor, color changes, and a slight decrease in light transmittance in the visible light region (400–800 nm) after the addition of LNPs, while the starch/lignin (SL) composite films exhibit excellent UV shielding properties, nontoxicity, mechanical properties (Figure 4), oxygen permeability, and good antioxidant performance.

Starch/lignin composite film production using an agro-industrial waste byproduct could assist in achieving waste biomass management in a cost-effective and environmentally friendly way. They are promising materials for slowing down the oxidative deterioration of soybean oil, food preservation, and the sustainable packaging industry. Kraft lignin has been blended in soy protein and glycinin, thermoplastic zein, wheat gluten, fish protein-based plastics, the protein matrix of wheat gluten, gelatine, soy protein, formaldehyde, and citronella essential oil to impart antifungal activity, etc.

Lignosulfonate, desulfonated lignosulfonate, and acid-hydrolyzed lignin have been introduced in the PLA matrix, leading to some important improvements in terms of mechanical and thermal stability. Lignin in polyhydroxy butyrate (PHB)-based biocomposites has also been used to improved properties. Lignin/PHB is completely biocompatible, biodegradable, and thermoplastic. The kraft lignin/PHB blend showed excellent biocompatibility and biodegradability.

All these applications of lignin are important reasons to consider lignin valorization in high-performance complex polymeric materials, as an important direction for sustainability and a circular economy. To date, studies on the interaction of lignin with the packaged food, as well as in vivo digestion, are very preliminary and will require additional investigation.

Generally, the compatibility of the lignin with polar components is a challenge; therefore, only small quantities of 10–25 wt% lignin can be used in PP or PE. Lignin can act as stabilizer for polyolefins, but when it is added to PS, the mechanical properties are deteriorated. Satisfactory results are obtained for blends with PET [325] or PVC.

Compostability of the lignin-based materials is an important aspect [326]. It has been mentioned above that lignin is efficiently biodegraded by white-rot fungi and various types of bacteria, but its degradation under composting conditions, commonly used to dispose of food packaging items, is incomplete and inefficient. The improved gas barrier, decreased water permeability, and increased hydrophobicity can be associated with reduction in the material degradability in the composting conditions.

Lignin biocomposites in biomedicine. Nanolignin (NL) and chitin nanofibril (CN) have been assembled into micro-complexes and loaded with bioactive factors, such as the glycyrrhetinic acid (GA) and CN-NL/GA (CLA). Completely biobased and bioactive films and fiber meshes designed for wound healing have been obtained from poly(3-hydroxybutyrate)/poly(3-hydroxyoctanoate-co-3-hydroxydecanoate), surface-modified via electrospraying/electrospinning of CN and NL and tested in vitro with human keratinocytes. The nanoparticles improved the antimicrobial and anti-inflammatory activity of the electrospun fiber meshes, leading to promise for wound healing applications [327].

**Table 5:** Lignin use in various green blends and composites, its role and applications (recent studies).

Components of Biocomposites	Lignin Role/Application of Product	References
Agriculture		
Azide-modified cellulose/0.5–2 wt% covalently bonded lignin with UV-blocking properties; propargylated lignin	Biodegradable, flexible, and transparent UV protection films from renewable resources	[297]
Polyvinyl alcohol PVA/lignin, spherical organosolv, lignin particles, LNPs obtained by dialysis with THF or ethanol as solvents	UV-blocking lignin–PVA composite film	[328]
Alkali lignin/carbon nanoparticles (C-NPs) containing lignin nanoparticles (L-NPs)	Agro-nanotechnology - Cost-effective alternative compared to conventional commercial fungicides - Environmentally benign nanopesticides for long-term plant protection - Antifungal nanocomposite - Control agent against *Fusarium verticillioides* in maize	[329]
Alginate/polyvinyl alcohol/calcium chloride and boric as crosslinkers	Three-dimensional (3D) biocomposite adsorbent; multiporous architecture; excellent substitute for a commercial EDTA-Fe micronutrient; eco-friendly, highly efficient	[330]
Waste treatment		
Hydrophilic sulfonated kraft lignin/polyethersulfone (PES)	Layer-by-layer (LbL) assembly; antifouling coating; oily wastewater treatment	[331]
Lignin-derived adsorption materials	Compounded lignin with other materials; high-value-added lignin adsorbent material wastewater treatment	[332]
Food Packaging		
Enzymatic hydrolysis of dried solid from alkali treatment of wheat straw/dry corn starch, glycerol, solution casting method	Lignin particles act as reinforcements for green biodegradable starch films	[323]
Technical lignin/cellulose	Cellulose/technical lignin composites; antioxidant and UV barrier Advanced packaging	[333]
Lignosulfonate/alginate	Photoprotective and antioxidant properties; enhanced barrier properties of the blend films and antioxidant activity; active packaging applications	[334]
Lignin nanoparticles/chitosan/polyvinyl alcohol	Lignin increased mechanical strength and antioxidant and antibacterial activity. Active food packaging	[335]
Low molar mass alkali lignin/PLA	Increased water barrier properties (up to 73%) photodegradability; biocompatibility, antimicrobial activity; very good cellular response and very low cytotoxicity levels; food packaging	[336]
Hydroxypropyl lignin or lignosulfonate or alkaline lignin/soy protein	Enhanced mechanical performance, water resistivity of soy protein plastics and specific functional properties; biomedical uses; food packaging	[337]
Alkaline lignin (AL) and sodium lignosulfonate (LSS)/PEG/thermoplastic zein	Strong H-bonding modifying α-helix, β-sheet, and β-turn secondary structures; improved physical properties; due to the interaction of AL with zein molecules, enhanced strength and water resistivity of soy protein plastics. Food packaging Biomedical uses	[338]
Lignin/tannic acid/biodegradable PBAT composite film: epoxidized soya bean (ESO) oil as plasticizer and encapsulated lignin/tannic acid as filler	Reduced water vapor and oxygen transmission rate; improved properties and degradability. Extended shelf-life and preservation of food, including fresh potato and onion Packaging of dry food products	[339]
Kraft lignin/poly(3-hydroxybutyrate) nano-composite Pickering emulsion and hot compression	Hydrogen bonding interactions; improved mechanical properties, UV resistance/blocking and higher melt viscosity; Food packaging	[340]
Technical soda lignin (ethyl acetate extract/high-density polyethylene. Melt extrusion	Composite for food packaging. Antioxidant and insect repellent activities	[341]
Biomedical		
Alkali lignin green synthesis of AgNP/carrageenan/calcium chloride, copper chloride magnesium chloride silver	Antibacterial against *Staphylococcus aureus and Escherichia coli*; biocompatible; Wound dressing and healing effect	[190]
Nanolignin (NL) and its composites	Cancer therapy drug and gene delivery, biosensing, bioimaging, and tissue engineering; therapeutic potency of chemotherapeutic drugs by decreasing their dose and reducing their adverse effects	[342]
Lignin as decoration for multi-walled nanotubes/PVA-lignin fiber nanocomposites	Antimicrobial properties Wound healing/tissue engineering	[343]
Lignosulfonate/PVA/chitosan	70% reduction in free radicals; good antibacterial abilities at 10% (*w*/*w*) lignin; wound healing	[220]
Kraft lignin/PVA/poly(glycerol sebacate) (PGS) electrospinning	PVA-PGS-lignin fibers. Lignin incorporation promotes neural cell proliferation and differentiation. Tissue engineering/regeneration	[344]
Kraft lignin/electrospun PCL fibers embedding lignin nanoparticles	Peripheral nerve regeneration	[345]
Alkali lignin/PLA-lignin nanofiber silver-ion-containing lignin nanoparticles/poly(lactide)–lignin nanofibers.	Antioxidant activity; eco-friendly alternative to AgNPs in antimicrobial and antioxidative applications; biomedical application Tissue engineering	[193]
Lignins extracted from pine residue esterified with succinic anhydride/PLA	Enhanced mechanical and thermal properties of PLA Excellent antimicrobial and biocompatibility.	[346]
Lignosulfonate/polyoxazoline/triazoles (linked silver)	Antimicrobial against many strains such as *Escherichia coli, Pseudomonas aeruginosa, Salmonella typhi Klebsiella pneumonia, Staphylococcus aureus, Staphylococcus epidermidis*, *Candida albicans*, and *Candida tropicalis*; Antioxidant, anti-inflammatory, preventing infection; promotion of healing; reduced inflammation on burn wound	[347]
Softwood kraft lignin/poly(butylene succinate) melt-mixing extrusion	Antimicrobial and antioxidant properties; resistance to adherence of the common nosocomial pathogen *Staphylococcus aureus*. Pharmaceutical/biomedical applications	[181]
New High-Performance Materials		
Organosolv lignin/polylactide/PVAc poly(vinyl acetate)/GMAglycidyl methacrylate, reactive extrusion	Super-toughened bio-elastomer PLA composite; improved interfacial adhesion; PVAc and GMA as toughening agents; single glass transition temperature. High elongation at break and impact strength; replaces petroleum-based elastomers such as EPDM elastomer	[348]
Micro- or nano-Soda lignin (NL) as green filler; content between 0.5–5 wt%/PLA masterbatch prepared by solvent casting, then melt mixing.	Biobased and biodegradable PLA films; Interfacial interactions, slightly stronger in the case of NL acting as fillers. Competitive green alternative in the food packaging industry	[201]
Acetylated lignin/thermoplastic polyurethane mixing	Lignin-based thermoplastic polyurethane adhesive. Wood adhesive	[349]
1–10 wt% grape seed lignin/highly crystalline (PHB)/amorphous (PHA). Polyhydroxyalkanoate modification	Improved mechanical and gas-barrier properties, high antioxidant capacity of lignin; biodegradability; active biodegradable packaging films; lignin can change the crystallinity of PHB in compost; nontoxicity of materials and its degradation products; positive effect on white mustard (*Sinapis alba* L.) seed germination	[350]
Methylated lignin, lignin esters, or lignin-containing epoxy groups/poly(butylene adipate-co-terephthalate) (PBAT) coextrusion; PBAT grafted with maleic anhydride as a stabilizer; poly-3-hydroxybutyrate PHB or poly-3-hydroxybutyrate-co-3-hydroxyvalerate	Economically competitive biodegradable PBAT/lignin and PHB/L composites Low-cost filler, 36% price reduction; good properties; degradability	[351]
Lignin-containing microfibrillated cellulose isolated from chemi-thermomechanical pulp or acetylated/PLA	Biocomposites with controlled biodegradation of polylactic acid (PLA). Biotic degradability	[352]
Unfunctionalized lignin and 3-aminopropyltriethoxy silane functionalized lignin/waterborne polyurethane. Chemical reaction	Lignin as natural reinforcing filler	[353]
Lignin/polyfurfuryl alcohol	Thermosets; Eco-friendly composite resins	[354]

Known applications of lignin-based composites are packaging, plant protection, biomedical materials, life sciences, automotive, advanced biocomposites, flame retardants, electroactive materials, energy storage, and others [355]. Synergistic interactions found in lignin-containing systems, mainly in nanocomposites and nanohybrid assemblies, are highly beneficial [356]. Most of the publications related to lignin-containing blends and composites used kraft or alkali lignins; especially kraft lignin in crude form. Higher risks in terms of cytotoxicity compared to lignins stemming from other biorefinery approaches like organosolv processes are possible.

## 10. Energy Storage and Conversion Technologies

Fossil fuels are increasingly used day-by-day worldwide, which will lead to their eventual depletion. The resulting environmental degradation necessitates the progression of renewable energy sources and energy storage/conversion technologies. Attempts have been made to seek out sustainable different energy sources. Plant biomass, especially wood containing mainly cellulose and lignin with a content of 18–30%, should be a good choice. Polymers and their composites play a vital role in energy storage and conversion technologies. A large volume of lignin is used as biofuels and in energy production. Lignin can partly replace fossil-based materials and allows more environmentally friendly formulations. Renewable lignin could reduce non-renewable coke consumption by approximately 20%.

### 10.1. Sustainable Fuel from Lignin

Sustainable energy sources and biofuels from renewable sources are very important. Bio-oils are rarely blended with crude oil as a feedstock for refineries. Lignin-derived fuels could reduce greenhouse gas emissions and dependency on fossil fuels. There are two basic methods for converting lignin into fuel: thermochemical and catalytic. Preliminary materials are utilized in hydrotreatment and fluid catalytic cracking (FFC) with zeolites as catalysts. Catalytic hydrogenation converts low-value products (as aromatics) to jet fuel. This coproduction improved the overall economic viability of an integrated biorefinery process [357,358,359,360].

### 10.2. Sustainable Aviation Fuel (SAF)

Sustainable aviation fuel (SAF) is essential to decreasing the carbon footprint of the aviation industry. A team in the USA, the National Renewable Energy Laboratory (NREL), Massachusetts Institute of Technology (MIT), and Washington State University used lignin for the production of the SAF. The two main challenges are finding an effective catalyst and removing the oxygen from the lignin. The lignin oils in existing research projects had an oxygen content of 27 to 34%. For aviation fuel, however, this value has to be lower than 0.5%. To date, catalysts containing expensive precious metals have been used, which, in addition, showed only low efficiency. Carbon dioxide emissions can only be achieved with the massive use of SAFs [361]. A collaboration of US researchers has pioneered a 100% sustainable aviation fuel using lignin [362].

### 10.3. Electrochemical and Energy Storage Applications

Because of its redox functionalities, lignin has an important role in various energy conservation processes where energy conversion and storage occur through a series of reversible oxidation/reduction reactions such as respiration and photosynthesis. The use of lignin in energy storage devices contributes to obtaining greener energy, improving performance, and also decreasing price and toxicity. Lignosulfonates are preferred for such applications [363]. Most studies deal with the improvement of electrochemical performance, novel lignin sources for this purpose, or structure and surface modifications of obtained materials. A process–structure–properties–performance correlation was associated with lignin valorization from a byproduct of biorefineries to high-performance energy storage materials, with low-cost production.

Lignin shows high potential for use as a renewable precursor for carbon materials, utilized in many energy storage devices. These applications include lignin as an expander for lead–acid batteries, electrodes for primary and rechargeable batteries, electronic double layer capacitors, and electrochemical pseudocapacitors as well as feeding different types of fuel cells, solar cells, etc. [364,365,366,367].

### 10.4. Lignin-Based Organic Flow Batteries

Organic flow battery systems for ships featuring electric propulsion have been developed in the maritime sector. One of the most promising technologies for this purpose is based on lignin developed by the German company CMBlu. Lignin-based organic flow batteries are more secure and stable, less costly, and more sustainable regarding resource use. Finally, the disposal is ecological, the components are easily recycled, and no problematic waste products are generated [368].

## 11. Conclusions

### 11.1. Lignin as Promising Renewable Source

The global demand for lignocellulosic biomass and for energy and chemical production has increased, along with the available amount of lignin as waste. Lignin valorization has been mainly focused on production of fine chemicals and biobased high-performance materials. The market size for lignin-based products includes colloidal lignin particles, vanillin, catechol, propyl guaiacol, BTX, PHA, PUR foams as polyols, carbon fibers, eugenol, and jet fuel. At the market level is established that, the selected applications depend on the lignin type. Low-purity lignin is utilized in energy production, bitumen, and biofuel, and with the increase in purity, other uses are envisaged, such as in production of the BTX, phenols, cement additives, activated carbon, and phenolic resins, while high-purity lignin is used to obtain carbon fibers, vanillin, phenol derivatives, materials with applications in medicine, pharmacy, cosmetics, and the food industry, etc. With increased lignin purity, their costs increased and also the value of the obtained products. Thermoset networks via step-growth polymerization from lignin-derivable compounds are also modern fields of research. From this renewable but heterogeneous chemical resource, greater value could be gained by developing higher value pharmaceutical applications which would help to improve integrated biorefinery economics.

A transition from the current petrochemical infrastructure requires either that: (1) regulations accelerate a shift from petroleum or (2) polymers derived from lignocellulosic biomass exhibit superior performance or longer-term (socio) economic advantages. The realization of cost-competitive petrochemical alternatives from biomass may require significant innovations in separation, purification, and polymerization chemistry. Bio-sourced materials are inherently greener than petrochemical-based systems, but the complexities are associated with lignin heterogeneity and modifications. The non-homogeneous, polydisperse nature and varying molecular weight and structure of lignin result from highly biodiverse sources and the lack of standardized extraction procedures, which is important when the lignin biomaterial is used for medical purposes such as in scaffold fabrication. Research interests are focused on the development of lignin nanoparticles/nanofibers, hydrogels, green composites as multicomponent, functional materials, etc., with a complex composition, which assures the good performance of materials through these synergistic interactions. The use of 3D-printable lignin monomers/formulations is particularly beneficial, but a lack of material diversity is considered a major limitation in stereolithographic printing. The high-volume utilization of lignin in 3D printing applications would further broaden its application fields and alleviate the environmental load of lignin. Lignin is one of the prime candidates for various biomaterial applications, such as raw materials, additives, drug and gene delivery, biosensors, bioimaging, 3D printing, tissue engineering, and dietary supplements. In applications related to food and pharmaceutical fields, the toxicity profiles require more in-depth, long-term analyses due to the phenolic nature of lignin biomaterials. Detailed structure/function relationships must be investigated to better understand the mechanism and behavior of lignin-drug carrier interactions, and also detailed studies on lignin biomaterial sterilization, in vivo biodegradation, and biocompatibility to better assess the role of lignin on cells and tissues are necessary.

The use of bulk lignin in composite materials incorporates renewability, reduces costs, and increases functionality. Novel bio-nanocomposites with improved properties and multifunctionality domain can be envisaged as an emerging, open field of research, with many possibilities because of the great abundance and diversity of biopolymers in nature, synergistic combination with inorganic nanosized solids, lignin graft copolymers, enzymatic grafting, and crosslinked networks.

### 11.2. Challenges and Future Trends

Lignin, the second-most abundant renewable biopolymer on Earth, is one of the largest natural renewable sources of aromatic structures. Sources of lignin are paper-pulping wastewater and agricultural/forestry residues, including straw, husk, stalks, stover, and cobs as the largest sources of wasted lignin, which are considered highly valuable as bioenergy and biorefinery materials because they are renewable, non-edible, and do not interfere with the food industry. Suitable valorization of lignin waste streams from the pulp and paper industry and biorefinery processes could be a crucial step for the development of a circular sustainable economy. The extraction of lignin is one of the major barriers in the reclamation and reuse of lignin from waste. Eco-friendly isolation of lignin from agricultural wastes is in the development stage and should be further studied [369]. Lignin has multiple impressive physicochemical properties (good mechanical and physicochemical properties, low weight, and excellent thermal stability, and it can undergo a range of modifications to tailor or impart special characteristics, such as improved compatibility and processability) and shows important bioactive effects (antioxidant, antimicrobial, antifungal, anti-inflammatory properties, etc.) being considered a multipurpose/multifunctional raw material with important roles/applications in the various fields. Therefore, lignin possesses huge potential for the production of a variety of materials because of its high carbon content, low cost, and bio-renewability. However, challenges appear because of the large differences found in molecular structure and molecular weight of lignins resulting/extracted from different sources (plant family and species, their culture conditions, part of plant, age, climate, etc.), extraction procedures, and after further treatments. Some technical/processed lignins are less chemically reactive and more cytotoxic compared to native lignin. In such cases, it is important to both evaluate in detail structure, molecular weight, biocompatibility, cytotoxicity, etc. of each type of lignin and to select suitable type for a certain application domain. Better-controlled composition and properties of the obtained products are necessary. The characterization and quantification of functional groups and the ratio of monomeric units are the main characteristics of technical lignins, affect the possibilities and strategies for further processing, and are among the chief obstacles of the utilization of this highly abundant biopolymer. Although several techniques were developed for this purpose, there is still a need for quick, cost-efficient, detailed characterization and reliable quantification methods for the utilization and modification of lignins for high-performance materials.

Because of its large number of hydrogen bonds, lignin has strong intermolecular and intramolecular forces, showing either favorable interactions with some components or a poor incompatibility and dispensability in complex polymer systems. To overcome these difficulties, careful selection of lignin type and modification methods for improving the compatibility have been used. Numerous studies have been conducted to develop natural alternatives, starting from biodegradable, renewable resources, which are safer and cleaner for the environment, including lignin valorization. New materials will be developed by combining green chemistry and engineering technology as a consequence of the demand for development of environmentally friendly and sustainable production/consumption/waste management systems for both plastics and biodegradable materials. This trend has changed in recent years because of modifications in consumer habits; preferences for single-use products, fresh food, and ready-to-eat packed food; awareness of the need for a healthy life and environment; and the growing need for fabricating packaging, medical devices, healthcare products, and others in a more environmentally friendly way. Lignin-based materials are excellent candidates for greenhouse gas diminution in comparison to many petroleum-based chemicals.

However, the valorization of lignin to high-performance and cost-competitive materials remains a challenge. It better adheres to ”green design” principles in comparison to petroleum counterparts, but its properties rank below those of several common petrochemical plastics with respect to environmental impact considering a full life-cycle analysis and economic gains. Moreover, lignin valorization has encountered a series of constraints related to its heterogeneous polymeric nature/composition, intrinsic recalcitrance, strong smell, dark color, some problems encountered in lignocellulose fractionation, recalcitrance to depolymerization/deconstruction, and a complex mixture of aromatic compounds resulting during degradation, etc. Deconstruction and chemical modifications will increase lignin utilization in complex environmentally friendly polymeric materials. However, these procedures require additional investments in the form of solvents, reagents, and energy inputs.

The simulation of biorefinery processes for the design of manufacturing processes to obtain value-added chemicals from lignocellulosic resources has been conducted, but this method must be continuously developed to assess/control all steps of the studied processes. Researchers and the industry should pay attention to life-cycle assessment (LCA) studies and technical–economic assessments. The importance of reactivity and constant quality of lignin as a raw material is also necessary. The developments are necessary in the alternative preparation methods to ensure low cost, biocompatibility, surface modification, sustainability, eco-friendliness and ease of large-scale production of LNPs and hybrid materials, especially those designed for medical applications. Functionalization of lignin to improve its polymerization with other polymeric materials is an open direction for research, and in vitro and in vivo detailed toxicity studies are required to facilitate the application of lignin-based hydrogels, LNPs, biocomposites in the food and pharmaceutical industries, and biomedicine. The large-scale utilization of lignin in 3D printing is still a great challenge due to its inherent brittleness and non-thermoplasticity. Cost-effective technology, scaling, and proof of concept are required to attract industrial production for lignin-based hydrogels/LNPs and green composite commercialization.

## Figures and Tables

**Table 1:** Characteristics of technical lignins adapted from [54,55,56].

Characteristic/ Technical Lignin	Soda and Alkali Lignin	Kraft Lignin	Acid Hydrolysis/ Enzymatic Lignin	Organosolv Lignin	Lignosulfonates	Ionic Liquid Lignin	Steam Explosion Lignin
Lignin separation	For non-woody biomass; 13–16% NaOH 140–170 °C	Na_2_S and NaOH 150–180 °C Thermochemical conversion of black liquor-small alkali soluble fragments	Diluted or concentrated acids as H_2_SO_4_, HCl, HNO_3_, H_3_PO_4_, maleic acid	Hydrothermal treatment of biomass with water and organic solvent	SO_2_ and water at 140–160 °C and carbonates and hydroxide salts	Ionic liquid	Mild hydrolysis; steam at high T temperature and pressure
Ash, %	0.7–2.3	0.5–3.0	1.0–3.0	1.7	4.0–8.0	0.6–2.0	5–8
Moisture content, %	2.5–5.0	3.0–6.0	4.0–9.0	7.5	5.8	-	
Carbohydrates, %	1.5–3.0	1.0–2.3	10.0–22.4	1–3	-	0.1	2.5–4
Acid soluble lignin, %	1.0–11	1–4.9	2.9	1.9	-	-	
Nitrogen, %	0.2–1.0	0.05	0.5–1.4	0–0.3	0.02	-	
Sulphur, %	0	1.0–3.0	0–1.0	0	3.5–8.0	1.5/	0–0.5
Phenolic hydroxyls, %	2.9–5.1	2.6–5	3–9	3.7–3.4	2–2.5	4.5–7	
Acids, %	5.4–13.6	4.1–6	6–10	7–8	4.6	1.0–5	
Methoxy, %	10–19	11–14	−19	−15	9	−13	
Purity	Moderate–High	High	Moderate–Low	High	Low–moderate	Moderate–Low	
Molecular weight, Mw	1000–3000 (up to 15,000)	1500–5000 (up to 25,000)	5000–10,000	500–5000	1000–50,000 (up to 150,000)	≈~2000	3500–15,000
Polydispersity	2.5–3.5	2.5–3.5	4.0–11.0	1.5–4.2	7.0	-	

**Table 3:** Characteristics of lignin-containing hydrogels and their applications in various domains (recent studies).

Hydrogel Components	Characteristics and Applications	Refs
Agriculture		
Alkali lignin and -based poly(ethylene glycol) diglycidyl ether	Increased availability and retention of soil water, which is beneficial for plant growth; high efficiency in severe dryness conditions; alleviated drought stress in maize	[231]
Physically crosslinked alkaline and organosolv lignins or lignins/poly(vinyl alcohol) hydrogels	Significant swelling and water holding capacity; addition of lignin enhanced the swelling/water retention ability of hydrogels by 800%	[232]
Lignin/alginate/konjaku flour-based hydrogel	Increased water holding/retention and of the nutrient retention capacity of the soil, increase in the mass of tobacco plant under dryness conditions	[233]
Lignin/polyacrylic acid-based hydrogels	Controlled release of some pesticides (i.e., paraquat, cyfluthrin, and cyhalofop-butyl)	[234]
Wastewater treatment and removal of pollutants		
Black liquor lignin-hydroxyethyl cellulose hydrogel	Super-absorbent hydrogel; removal of dye pollutants	[235]
Polyacrylic acid-g-pretreated alkali lignin porous hydrogel	Removal of Pb^2+^, Cu^2+^, and Cd^2+^	[236]
Lignin functionalized with hexadecyltrimethylammonium-bromide-based spherical particles	Lignin-based biosorbents; removal of vanadium (V) ions from aqueous solution	[237]
Lignosulfonate/lysine hydrogel	Adsorption of heavy metal ions	[238]
Lignin/PVA or LNP/nanocellulose (cryogels); anchoring lignin nanoparticles (LNPs) to the nanocellulose network via electrostatic attraction	Environmental engineering. Adsorbents for pharmaceutical pollutants (e.g., diclofenac, metoprolol, tramadol, carbamazepine) and Bisphenol A	[186,239]
Hydrogel was obtained by free-radical polymerization in the presence of methylenebisacrylamide (MBA), alkali lignin (AL), and potassium persulfate (PPS). Carboxylated cellulose nanofibrils (CCNs) and carbon dots (CDs) as fluorescent probes were prepared via a condensation reaction	Fluorescent hydrogel. Adsorption of Cr (VI), excellent optical properties, biocompatibility, and nontoxicity significant efficiency in adsorption and detection of Cr (VI)	[240]
Biomedical [206]		
Alkaline and organosolv lignins, hydoxymethylated lignins, peroxidated lignins/PVA	Onion peels were valorized for quercetin (natural drug) microwave extraction; controlled drug delivery, and quercetin-controlled delivery applications	[241]
Lignin-agarose hydrogel/silk fibroin (SF) embedded zinc chromite nanoparticles	Hemocompatibility and antibacterial activity; complete healing of the wounds in mice treated with the scaffold of crosslinked lignin–agarose/SF/ZnCr_2_O_4_ nano-biocomposite after five days; wound healing application;	[242]
Enzymatic hydrolysis lignin (wheat straw)–alginate cryogels and cryogels obtained from the freezing technique; wet and dry alginate–lignin aerogels	Porous structure; lignin reduced hydrophilicity of alginate; wound healing and tissue repairing/tissue engineering; regenerative medicine	[243]
Lignin-co-gelatin (cryogels) and chemically crosslinking and Ag_2_O/CuO NPs	Antioxidant, antibacterial, injectable lignin/gelatin composite cryogels; wound healing and tissue engineering; good mechanical properties and microporous structure; very good free scavenging activity; inhibited growth of both Gram-positive and Gram-negative bacteria	[244]
Lignin/gelatin hydrogels with different concentrations of lignin	Fast-release drug carriers used to deliver Ribavirin in COVID-19 treatment	[245,246]
Organosolv lignin/gelatin composite cryogels obtained by chemically crosslinking at −20 °C. Organosolv lignin and gelatin; multifunctional, bioactive lignin-co-gelatin composite cryogels obtained by chemically crosslinking lignin together with gelatin at −20 °C.	Multifunctional, bioactive cryogel with antimicrobial (reduced *E. coli* and *S. aureus* viability) and antioxidant activities; in vitro cyto-compatibility (after 72 h of incubation, viability of 3T3 cells was 97%); improved mechanical properties and syringe injectability	[219,242]
Ag/lignin NPs/PAA-pectin hydrogel	Good mechanical, antibacterial, and wound healing properties; epidermal growth factor loaded in the Ag-lignin NPs-PAA-pectin hydrogel increases the wound healing activity	[188,247]
Lignin/poly(ethylene) glycol diglycidyl ether hydrogel	Drug delivery; controlled release of paracetamol	[248]
Organosolv lignin extracted from coconut husks/polyethylene glycol (PEG), polypropylene glycol (PPG), polydimethylsiloxane (PDS) nanogel	Thermo-responsive polyurethane-based copolymer nanogel; lignin-incorporated nanogel; acts as an antioxidant biomaterial for wound healing; human L-02 hepatocyte cell viability > 90%; rapid and complete wound healing after 25 days; wound-dressing application	[249]
Lignin-carbohydrate complex/PEG diglycidyl ether	Cell carriers; positive hepatocyte adhesion; biocompatibility	[250]
Alkali lignin/chitosan hydrogels	Wound healing applications; low cytotoxicity, biocompatibility, 99 ± 3% cell viability	[143]
Lignosulfonate/poly(vinyl alcohol)/chitosan composites hydrogel; lignin mixing an aqueous acidic solution of chitosan and solutions of lignin and PVA	Composite hydrogel; 10 wt% lignin had enhanced hydrophilicity, antimicrobial behavior, and antioxidant capability; wound dressing	[220]
Lignosulfonate; in situ reduction of AgNPs then crosslinked in the lignin/PVA hydrogel	Improved antibacterial activity against *Staphylococcus aureus* and *Escherichia coli*, non-cytotoxicity, biocompatibility, wound healing	[251]
Lignin/PVA cryogel; freeze-drying crosslinking and curing methods	“Smart” biomaterial scaffolds elaborated by using a factorial design scaffold fabrication model; 800% water retention capacity; controlled drug loading and delivery; pH and temperature responsiveness; antifungal activity against *Aspergillus niger* strain	[232,252]
40–24% lignin-containing alcohol groups/Gantrez S-97 (GAN) (methylvinylether and maleic acid copolymer)/PEG by esterification reaction accelerated by microwave radiation, crosslinking time was reduced from 24 to 1 h	Reductions in *Staphylococcus aureus* and *Proteus mirabilis* adherence (two common pathogens responsible for medical-device-associated infections); curcumin delivery	[222]
Glycinated kraft lignin/hyaluronic acid hydrogels	Non-cytotoxicity (>90% cell viability); positive cell migration and cell growth	[253]
Kraft lignin/PLA/Rose Bengal, gold, silver	pH-responsive hydrogels; antifungal and antimicrobial properties at very low IC50 values (0.1 μg/mL)	[254]
Lignin epoxy-modified resin/xanthan crosslinking with epichlorohydrin	Superabsorbent hydrogel; high swelling rate in aqueous medium; release of hydrophilic bisoprolol fumarate drug for high blood pressure and heart failure treatments	[255]
Low and high lignin content/cellulose nanofiber; ultrasound-assisted	Tissue engineering; cytocompatibility with gingival fibroblast cells	[256]
Methacrylated lignin (lignin-MA)/sulfobetaine methacrylate (SBMA) double network structure consisting of chemical and physical crosslinking between SBMA and lignin-MA	Lignin-MA as hydrogel skeleton and antibacterial agent; 94.8% reduction rate against *E. coli* and 95.7% against *S. aureus;* high hydrophilicity because of zwitterionic SBMA. Antifouling and antimicrobial properties, potential applications in medical devices and as biological material	[257]
Food packaging		
Lignin (black liquor of bagasse) into PVA/gelatin blends	Antimicrobial and antibiofilm activities against food-borne contaminants as Gram-positive bacteria *Bacillus subtilis* and *Staphylococcus aureus* and Gram-negative bacteria *Escherichia coli* and *Pseudomonas aeruginosa*	[258]

**Table 4:** Recent examples of lignin use in 3D printing materials, their properties, and applications.

Components	Characteristics and Applications	Refs
15% by weight modified lignin-containing photoactive resins	These materials generate new products for additive manufacturing applications, with a four-fold increase in ductility. Excellent print quality was obtained with modified lignin resins with a commercial stereolithography system	[288]
Organosolv lignin OSL/hydroxypropyl cellulose (HPC), 40/60 and 50/50 mixtures	Lignin/HPC biobased aqueous inks; direct-write lyotropic blends; high performance	[289]
Lignin–carrageenan/AgNP-MgCl_2_ one-pot synthesis of AgNPs	Lignin as a reducing agent for AgNP green synthesis and capping agent in the carrageenan matrix crosslinked with divalent cations; wound healing; fast wound dressing	[290]
Spherical colloidal lignin particles/cellulose nanofibril-alginate	Three-dimensional printed cell culture model; soft-tissue engineering	[291]
Keratin/lignin hydrogels	Smooth films and nanoparticles; 4D functional biocomposite materials. Protein complexation by lignin was applied to form copolymers and reinforce keratin crosslinking networks on aqueous and solid state processing 3D and 4D printing	[292]
Kraft lignin/keratin	Fully biodegradable keratin hydrogels through ”greener” processes. Lignin binder to deliver 3D-printability; keratin/lignin biocomposite materials. Lignin as material additive manufacturing technology: 3D and 4D printed responsive materials without the need for synthetic chemical modifications	[293]
Granular dealkaline lignin/corn-derived zein protein by extrusion	3D-printed insoluble lignin granules act as a binder for enhanced printability and degradation in soil. Suitable biocomposites for 3D printing manufacturing biodegradable circuit boards are used, along with inexpensive industrial byproducts lignin and zein as precursors and benign solvents, such as ethanol and water	[294]
Poly(caprolactone)/lignin/loaded curcumin	3D-printed dressings; antioxidant and antimicrobial activities	[286]
Carboxylated lignin/polylactic acid melt mixing;	Suitable biocomposites for 3D printing via fused deposition modeling (FDM); improved interfacial adhesion between the COOH-lignin surface and PLA matrix; reduced cost of printing PLA 3D filaments without changing their thermal and mechanical properties	[295]
Lignin-coated cellulose nanocrystal/methacrylate composites	3D stereolithography printing; mechanical reinforcement and thermal stabilization	[276]
